# An Introduction to Traditional Healing in American Indian and Alaska Native Communities

**DOI:** 10.15766/mep_2374-8265.11506

**Published:** 2025-03-07

**Authors:** Alec J. Calac, Hailey A. Baker, Daniel J. Calac

**Affiliations:** 1 Third-Year Medical Student, University of California, San Diego, School of Medicine; 2 Fourth-Year Medical Student, University of Minnesota Medical School; 3 Chief Medical Officer, Southern California American Indian Health Center; † Tribal affiliation: Pauma Band of Luiseño Indians; ‡ Tribal affiliation: Cherokee Nation

**Keywords:** American Indian or Alaska Native, Cultural Humility, Indian Health Service, Indigenous, Native American, Traditional Healing, Community-Based Health Care, Complementary/Alternative Medicine, Cultural Competence, Health Equity, Spirituality, Well-Being/Mental Health, Diversity, Equity, Inclusion

## Abstract

**Introduction:**

The United States has a trust responsibility to provide health care to members of the 574 federally recognized American Indian and Alaska Native (AI/AN) Tribes and Villages through the Indian Health Service, an agency tasked with promoting AI/AN health and cultural connectedness. Despite the presence of a comprehensive health care system in 37 states, physicians and allied health professionals receive minimal health professional education regarding the sociocultural factors affecting AI/AN health. This module addresses the underrepresentation of AI/AN health professional curricula and promotes a greater understanding of AI/AN health determinants and cultural constructions of health for individuals with limited exposure to these topics.

**Methods:**

We developed a 60-minute interactive session aimed at increasing trainees’ understanding of AI/AN traditional healing practices across the medical education continuum. The session consisted of a PowerPoint presentation, one video, and multiple small-group discussion exercises. The session was evaluated with pre- and postsurveys and implemented four times at medical school seminars.

**Results:**

There were 37 respondents in total. Analysis of pre/post survey responses to confidence in meeting each learning objective showed a significant increase in confidence for each of the three learning objectives (*p* < .01). Respondents were very interested in how traditional healing improved health intervention outcomes and showed interest in connecting AI/AN patients to these services.

**Discussion:**

This module's positive reception indicates that it can serve as an important educational tool for learners involved in AI/AN-focused clinical care. Learners were able to explain how traditional healing practices are important in promoting AI/AN health.

## Educational Objectives

By the end of this activity, learners will be able to:

1. Describe why traditional healing practices are important in American Indian and Alaska Native communities.
2. Review the literature that supports the integration of traditional healing practices with medical services in American Indian and Alaska Native communities.
3. Identify demonstration projects that assess the impact of traditional healing practices on health outcomes in American Indian and Alaska Native communities.

## Introduction

There is a poor understanding of the social and structural determinants of American Indian and Alaska Native (AI/AN) health and wellness in academic medicine. The Association of American Medical Colleges estimates that only 11% of MD-granting institutions have AI/AN health curricula for learners.^1^ Similar data are not available from the Association of Colleges of Osteopathic Medicine, which represents DO-granting institutions. Complicating this, it is estimated that less than 1% of medical students, practicing physicians, and full-time medical school teaching faculty identify as AI/AN, which is far less than their representation of 3% in the general United States population.^1,2^ In some states, the proportion of the AI/AN population ranges up to 20%, underscoring the need to train a workforce responsive to AI/AN communities’ unique needs.^2^

One such aspect of AI/AN health not adequately included in medical education is traditional healing. There are 574 federally recognized AI/AN Tribes and Villages in 37 states, each with their own unique history, culture, and relationship with the federal government.^3^ The Indian Health Service (IHS), an agency within the US Department of Health and Human Services, is responsible for providing health care to tribal members.^3^ The IHS's mission is to raise the physical, mental, social, and spiritual health of AI/AN patients to the highest level.^4^ Traditional healing services may be important to AI/AN patients, particularly elders; thus, it is important to provide health care providers with a fundamental understanding of AI/AN traditional healing services and the role of traditional healers within the physician-led health care team. Such efforts may work to redress centuries of intergenerational trauma and medical mistrust among the AI/AN population and promote greater coordination of care between the health care team and traditional AI/AN healing figures, colloquially referred to as medicine men or women or by culturally specific terms in their respective communities (e.g., hatááłii, medicine men in the Navajo Nation).^5,6^

Traditional healers provide special attention to the mental, spiritual, and social needs of AI/AN individuals and communities and are held in high regard for their knowledge and role in precontact and contemporary AI/AN societies.^7^ Today, there is a paucity of literature describing the importance of traditional healers, which is partly due to the privacy of these practices within AI/AN communities and safeguards against appropriation of traditional medicines and healing practices. However, the importance of these practices should not be discounted. In 2022, the White House Office of Science and Technology Policy published government-wide guidance recognizing and including Indigenous Knowledge in federal research, policy, and decision-making.^8^ This guidance calls attention to the principle that Indigenous Knowledge and other forms of knowledge, such as Western methods of scientific inquiry, do not depend on each other for validation and that the systems can support, rather than work against, each other.

Existing modules in *MedEdPORTAL* on complementary and alternative medicine do not focus on traditional healing practices in AI/AN communities. To educate health professionals on AI/AN health, there are two modules in *MedEdPORTAL* that identify the social and structural determinants of urban AI/AN health in Los Angeles, California, and barriers and facilitators for health systems delivery in two IHS hospitals based in South Dakota.^9,10^ These modules help learners understand the unique structure of the IHS as well as how state and federal policy affect AI/AN health. When coupled with our module, trainees can gain a better appreciation of culturally responsive care within the IHS and how physicians and members of the health care team can mitigate bias and discrimination in health systems serving AI/AN patients by coordinating care with traditional healers. It has been established that AI/AN and Indigenous patients seek health counsel from both traditional healers and physicians.^7^ However, they may not feel comfortable disclosing this information to their physician-led health care team due to medical mistrust, among other factors.^11^ Our interactive module, intended for medical students, as well as other allied health professionals-in-training, presents strategies and important background information to educate trainees about coordination of care between traditional healers and physicians, ensuring that the physical, mental, social, and spiritual needs of AI/AN patients are fully met. The module also incorporates a series of teaching methods, such as didactic learning, small-group discussion, and teach-back instruction, so that trainees leave with a greater understanding of the importance of traditional healing in AI/AN communities. Additionally, we challenge learners to appreciate a multilevel understanding of the social and structural determinants of AI/AN as presented in a National Institute of Minority Health and Health Disparities research framework adapted to reflect historic and sociocultural influences on AI/AN health.^4^

## Methods

This module was drafted, implemented, and evaluated by a team composed of AI/AN researchers and health care professionals. The primary team consisted of Daniel J. Calac, an AI/AN board-certified physician-educator and researcher; Alec J. Calac, an MD/PhD candidate at an academic medical center, researcher, and health policy advocate; and Hailey A. Baker, an MD candidate at an academic medical center, researcher, and public policy fellow. They were advised by John Paul Sánchez, a physician and health workforce researcher with previous AI/AN-focused research experience with a nonprofit organization, and Kaitlyn Pommells, an academic medicine writing coordinator at a nonprofit organization. Feedback was also sought from AI/AN physicians and traditional healers to improve the quality of the material presented.

The module was facilitated four times by Alec J. Calac and Hailey A. Baker, once at an academic medicine conference sponsored by Building the Next Generation of Academic Physicians (BNGAP) and by invitation at three medical schools and academic medical centers, including the University of California, San Francisco, School of Medicine; the University of New Mexico Health Sciences Center; and the Eastern Virginia Medical School. The module was primarily presented in November 2023 because of that month's historic designation as Native American or American Indian and Alaska Native Heritage Month. Multisite institutional review board approval was provided by the University of New Mexico and accepted by the hosting institutions.

The focus of the module was to educate trainees about fundamental aspects of traditional healing in AI/AN communities. Information for delivering this module was presented in the facilitator guide (Appendix A) and could also be enhanced when working with AI/AN physicians and allied health professionals. The intended audience for this activity was medical students and allied health trainees who might be unfamiliar with how AI/AN patients accessed and navigated different sources of care, both in the community and in health care systems.

The module alternated between didactic learning slides and interactive small-group or self-reflective exercises aimed at growing learners’ understanding of the importance of AI/AN traditional healing practices, with detailed instructions presented in the facilitator guide (Appendix A), and maximizing opportunities for engagement. The PowerPoint (Appendix B) started with facilitator and learner introduction and a primer on terminology and language (acknowledging the heterogeneity of Indigenous and AI/AN cultures), followed by a visual presentation of how AI/AN traditional healing services were delivered by the federal agency and other stakeholders. The module concluded with a series of exercises encouraging learners to change their own clinical practice and engagement with AI/AN traditional healers as future or current members of the health care team.

We included a short 5-minute video on slide 18 (Appendix B) and other multimedia with Indigenous patients and traditional healers to educate trainees about what, why, and generally how traditional healing was important, acknowledging that these practices might be different from community to community. Other figures also described a nonexhaustive list of what traditional healing might entail and the significance of these practices. Our intention was to develop trainees’ cultural humility towards AI/AN and other diverse populations using complementary and alternative medicines and practices.

The following materials were required to successfully deliver the module. Facilitators needed to take an adequate amount of time, estimated by us as spanning approximately 2 hours depending on baseline knowledge, to review all materials and familiarize themselves with the content.

The facilitator guide (Appendix A) included pertinent background information for each slide as well as suggestions for delivery. It was necessary in preparaing for delivery of the module as well as being a key resource for those without expertise in AI/AN health. While we recommend including an AI/AN or Indigenous facilitator when using this module, it is not necessary to do so. However, the facilitator(s) should have a moderate or proximal background of reciprocal engagement in academic medicine and the health sciences with AI/AN-stakeholders. This is because we believe that delivering AI/AN health content is not a responsibility just of Indigenous Peoples but of all members of academic medicine across the allopathic and osteopathic medical education continuum.

The PowerPoint (Appendix B) was intended to guide the module and fulfill the educational objectives above. Consisting of 38 slides, all videos and supplementary materials were included within the PowerPoint. To assess module responsiveness to the learning objectives, learners were instructed to complete both a presurvey (Appendix C) and a postsurvey (Appendix D), each of which took no longer than 5 minutes. The evaluation data assessed trainees’ competency and personal and professional growth with AI/AN traditional healing and related topics after module delivery. All learners were optionally asked about their basic demographics (e.g., race, gender identification, age range, sexual orientation, and current professional one). Trainees rated their confidence to each objective using a 5-point Likert scale (1 = *not confident,* 5 = *completely confident*) both before and after the module. Trainees were also asked knowledge questions from Appendix D, using a 5-point Likert scale (1 = *not at all knowledgeable,* 5 = *very knowledgeable*) or a series of yes/no/unsure questions.

To deliver this module, facilitators had to have access to a computer and audiovisual equipment. The module duration was intended to span a 60-minute period but could be adjusted according to facilitator preference.

## Results

This workshop was implemented three times virtually with preclinical medical students at (1) the BNGAP Academic Medicine Writing Fellowship, (2) University of New Mexico Health Sciences, and (3) Eastern Virginia Medical School, due to constraints of the COVID-19 pandemic, and in person at the University of California, San Francisco, School of Medicine. Pre- and posttests were administered to 37 participants, of whom 25 completed both forms (68% response rate) and were included in statistical analysis. Demographic data, such as respondent race and level of training, were lost during analysis and were therefore unavailable for review.

Participants self-assessed their confidence in their ability to meet the workshop's learning objectives using a 5-point Likert scale (1 = *not confident,* 5 = *completely confident*). Analysis of pre- and posttest responses showed a statistically significant increase in confidence for each of the three learning objectives using a related-samples Wilcoxon signed rank test (Table 1). Using SPSS version 29.0, significance was achieved at the level of *p* < .01 for the comparison of each learning objective. Results of two descriptive questions assessing the participants’ knowledge demonstrated improvement in the average score between the pre/post tests (Table 2).

**Table 1.**
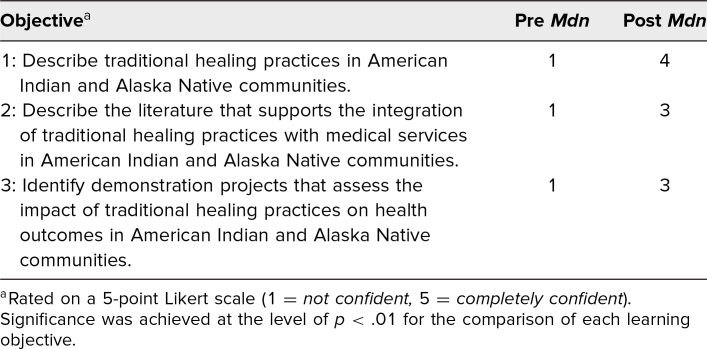
Pre- and Postworkshop Responses to Confidence Questions (*n* = 25)

**Table 2.**
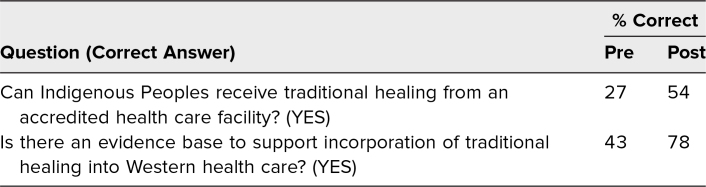
Pre- and Postworkshop Responses to Knowledge Questions (*n* = 25)

Nineteen participants commented that what they appreciated most about the workshop was the historical context and updated evidence/statistics. Two notable quotes were “The historical context and the updated status of care for AN/AI were very insightful” and “Providing context and evidence in the setting of the healthcare disparities will be useful in starting conversations and/or continuing dialogue.” Additionally, several respondents mentioned that the presentation was informative and interactive. Thirteen participants provided quality feedback about what should be done to improve the workshop. This feedback included suggestions to add additional examples or descriptions of AI/AN traditional healing practices in community-based or clinical settings, such as an article or video. Other feedback asked for additional learner engagement strategies and self-directed resources for learners interested in learning more about traditional healing, as well as how to incorporate traditional healing into an AI/AN patient's social and medical history.

## Discussion

This is one of the first educational modules in medical education focusing on the importance of traditional healing practices in AI/AN communities. It is important that all physicians and allied health professionals understand the social and structural determinants of AI/AN health because the majority of the AI/AN population lives away from their respective tribal lands and may not have access to the IHS.^12^ By fostering cultural humility and cultural safety in an AI/AN-focused context, health care providers can engender trust with AI/AN patients and encourage healing, rather than trauma.^13,14^ This module is an important step towards AI/AN health equity and the provision of safe, comprehensive health care services to a population that has prepaid for their health care through treaties and other agreements with local, state, and federal governments.

This module captures the survivance and resilience of AI/AN cultures as well as how traditional healing practices can promote AI/AN health and wellness. We often see AI/AN health disparities presented to learners without context, leading audiences to believe that genetic or behavioral factors must be the root cause of AI/AN hardship.^15^ This kind of presentation may perpetuate negative AI/AN stereotypes and cause harm to the population.^16^ Rather than doing this, we challenge learners to explain how policies and systems have perpetuated AI/AN health disparities and how traditional healing practices and cultural connectedness can improve AI/AN health and wellness.^15,17^ This was best observed during the facilitated discussions, when learners worked together to build a better understanding of AI/AN traditional healing. Based on discussion-related feedback, we also understood that learners valued a nonjudgmental atmosphere, especially for topics that they may not have been familiar with, so we modified some of the discussions to be self-reflective or small-group discussions. After reviewing respondent feedback for the entire module, we also modified our learning objectives to better capture the module's goals as well as to engage learners across multiple domains of knowledge.

Using the literature and narratives presented in this module, we hope to start a conversation about how coordination of care between physicians and traditional healers can improve the patient-physician relationship and also to encourage health systems and policymakers to remove structural barriers to accessing these services. Cultural connectedness and identification are important determinants of AI/AN health, especially in adolescence and young adulthood.^18,19^ They have also been shown to improve medication-assisted therapy outcomes for opioid use disorder, such as program retention and long-term abstinence.^20^ Several states, such as Arizona and California, have recognized these studies and are now asking the US Centers for Medicare and Medicaid Services for approval to pay for AI/AN traditional healing services at IHS facilities.^21,22^ In Arizona, a state with a large AI/AN population and health facilities using traditional healers, Medicare officials have worked with AI/AN leaders on model guidelines for incorporating traditional healing into the clinical setting, such as by defining eligible services and outlining how health systems can integrate AI/AN traditional healers into their facilities.^21,23^

There is limited written and publicly available research into the delivery and efficacy of traditional healing in tribal communities. In addition, AI/AN Tribes and Villages have unique practices that may be closed to those not from within the community. We are unable to fully encompass the breadth of traditional healing practices in this module and instead focus on overarching principles that can be generally applied when caring for AI/AN and Indigenous patients. Thus, we wish to make clear that our evaluation approach focuses on assessing trainees’ attitudes and beliefs about AI/AN traditional healing services and their future willingness to coordinate care with a traditional healer, not the cultural and spiritual benefits of these services in a health care setting.

We especially value the constructive feedback provided by participants. Respondents asked for additional examples or descriptions of AI/AN traditional healing practices in community-based or clinical settings. We added several AI/AN community-specific examples of traditional healing to enhance learners’ understanding of these practices as well as additional resources at the end of the module. Secondly, it was brought up that large-group discussions can be intimidating for some learners, especially those from marginalized backgrounds. We acknowledged this and restructured the facilitator guide with additional audience engagement strategies for each question and case-based scenario. We also added suggestions for the use of a real-time anonymous Q&A resource. Finally, we were encouraged that learners came away with a desire to change their clinical practice to better meet the needs of AI/AN patients. After consulting with AI/AN physicians and traditional healers, we also updated the presentation to include questions that medical students and physicians can ask their AI/AN patients about traditional healing and how this information can be properly documented.

Future directions include development of additional modules encompassing further aspects of AI/AN traditional healing practices, including, but not limited to, grief management, substance use disorder treatment, elder care, and palliative care. These modules may also spur greater development of AI/AN health professional curricula benefitting AI/AN communities, such as collaborations between academic medical centers and AI/AN communities.

## Appendices


Facilitator Guide.docxInstructional Slides.pptxTrainee Presurvey.docxTrainee Postsurvey.docx
*All appendices are peer reviewed as integral parts of the Original Publication.*

## References

[1] Reshaping the Journey: American Indians and Alaska Natives in Medicine. Association of American Medical Colleges; 2018. Accessed February 13, 2025. https://store.aamc.org/reshaping-the-journey-american-indians-and-alaska-natives-in-medicine.html

[2] Sánchez-Rivera AI, Jacobs P, Spence C. A look at the largest American Indian and Alaska Native tribes and villages in the nation, tribal areas and states. United States Census Bureau. October 3, 2023. Accessed February 13, 2025. https://www.census.gov/library/stories/2023/10/2020-census-dhc-a-aian-population.html

[3] About IHS. Indian Health Service. Accessed February 13, 2025. https://www.ihs.gov/aboutihs/

[4] Manson SM. NIMHD minority health and health disparities research framework: adapted to reflect historic and socio-cultural influences for American Indian and Alaska Native Nations. National Institute on Minority Health and Health Disparities. Accessed February 13, 2025. https://www.nimhd.nih.gov/about/overview/research-framework/adaptation-framework.html

[5] Pacheco CM, Daley SM, Brown T, Filippi M, Greiner KA, Daley CM. Moving forward: breaking the cycle of mistrust between American Indians and researchers. Am J Public Health. 2013;103(12):2152–2159. 10.2105/AJPH.2013.301480 24134368 PMC3828980

[6] Kahn CB, James D, George S, et al. Diné (Navajo) traditional knowledge holders’ perspective of COVID-19. Int J Environ Res Public Health. 2023;20(4):3728. 10.3390/ijerph20043728 36834423 PMC9964790

[7] Redvers N, Blondin B. Traditional Indigenous medicine in North America: a scoping review. PLoS One. 2020;15(8):e0237531. 10.1371/journal.pone.0237531 32790714 PMC7425891

[8] Indigenous Knowledge guidance for federal agencies. Bureau of Indian Affairs. November 30, 2022. Accessed February 13, 2025. https://www.bia.gov/events/indigenous-knowledge-guidance-federal-agencies

[9] Garcia AN, Castro MC, Sánchez JP. Social and structural determinants of urban American Indian and Alaska Native health: a case study in Los Angeles. MedEdPORTAL. 2019;15:10825. 10.15766/mep_2374-8265.10825 31161137 PMC6543927

[10] Tobey M, Sacks C, Foster D, Norman D, Karol S, Lee P. American Indian health in South Dakota—a health systems case. MedEdPORTAL. 2014;10:9869. 10.15766/mep_2374-8265.9869

[11] Jaramillo ET, Sommerfeld DH, Haozous EA, Brunner A, Willging CE. Causes and consequences of not having a personal healthcare provider among American Indian Elders: a mixed-method study. Front Public Health. 2022;10:832636. 10.3389/fpubh.2022.832626 PMC8926165 35309185

[12] Urban Indian Health Commission. Invisible Tribes: Urban Indians and Their Health in a Changing World. Urban Indian Health Commission; 2007. Accessed February 13, 2025. https://www2.census.gov/cac/nac/meetings/2015-10-13/invisible-tribes.pdf

[13] Allice I, Acai A, Ferdossifard A, Wekerle C, Kimber M. Indigenous cultural safety in recognizing and responding to family violence: a systematic scoping review. Int J Environ Res Public Health. 2022;19(24):16967. 10.3390/ijerph192416967 36554846 PMC9779508

[14] MacLean TL, Qiang JR, Henderson L, et al. Indigenous cultural safety training for applied health, social work, and education professionals: a PRISMA scoping review. Int J Environ Res Public Health. 2023;20(6):5217. 10.3390/ijerph20065217 36982126 PMC10049537

[15] Gampa V, Bernard K, Oldani MJ. Racialization as a barrier to achieving health equity for Native Americans. AMA J Ethics. 2020;22(10):E874–E881. 10.1001/amajethics.2020.874 33103650

[16] Chowkwanyun M, Reed AL Jr. Racial health disparities and Covid-19—caution and context. N Engl J Med. 2020;383(3):201–203. 10.1056/NEJMp2012910 32374952

[17] Zestcott CA, Spece L, McDermott D, Stone J. Health care providers’ negative implicit attitudes and stereotypes of American Indians. J Racial Ethn Health Disparities. 2021;8(1):230–236. 10.1007/s40615-020-00776-w 32445056

[18] Henson M, Sabo S, Trujillo A, Teufel-Shone N. Identifying protective factors to promote health in American Indian and Alaska Native adolescents: a literature review. J Prim Prev. 2017;38(1-2):5–26. 10.1007/s10935-016-0455-2 27826690 PMC5313316

[19] Brockie TN, Campbell JC, Dana-Sacco G, et al. Cultural protection from polysubstance use among Native American adolescents and young adults. Prev Sci. 2022;23(7):1287–1298. 10.1007/s11121-022-01373-5 35641730 PMC9489542

[20] Venner KL, Donovan DM, Campbell ANC, et al. Future directions for medication assisted treatment for opioid use disorder with American Indian/Alaska Natives. Addict Behav. 2018;86:111–117. 10.1016/j.addbeh.2018.05.017 29914717 PMC6129390

[21] Arizona's Section 1115 Waiver Renewal Request (2022–2027). Arizona Health Care Cost Containment System. Accessed February 13, 2025. https://www.azahcccs.gov/Resources/Federal/waiverrenewalrequest.html

[22] Tribal and Indian Health Program Representatives Meeting. California Department of Health Care Services; 2022. Accessed February 13, 2025. https://www.dhcs.ca.gov/Documents/Tribes-and-Indian-Health-Program-Representatives-Meeting-Presentation-02-24-22.pdf

[23] Kim C, Kwok YS. Navajo use of native healers. Arch Intern Med. 1998;158(20):2245–2249. 10.1001/archinte.158.20.2245 9818804

