# Supplementary material for: An Introduction to Traditional Healing in American Indian and Alaska Native Communities
Source: MedEdPORTAL. 2025 Mar 7;21:11506. doi: 10.15766/mep_2374-8265.11506 (PMC11885593; doi:10.15766/mep_2374-8265.11506)
Supplement: Supplementary file 1 — Facilitator Guide.docxInstructional Slides.pptxTrainee Presurvey.docxTrainee Postsurvey.docx [file mep_2374-8265.11506-s001.zip › A. Facilitator Guide.docx]

**An Introduction to Traditional Healing in American Indian and Alaska Native Communities**

**Overall Goals**

The United States (U.S.) has a trust responsibility to provide culturally-appropriate health care to American Indian and Alaska Native (AI/AN) patients. Physicians and allied health professionals are not likely to receive instruction about how AI/AN patients access different kinds of care, such as services provided by a traditional healer. The overall goals of this module are to introduce traditional healers as important members of the physician-led health care team for AI/AN patients and explain how the health care team can coordinate medical care with traditional healing services.

**Workshop Objectives**

1. Describe traditional healing practices in American Indian and Alaska Native communities;
2. Describe the literature that supports the integration of traditional healing practices with medical services in American Indian and Alaska Native communities
3. Identify demonstration projects that assess the impact of traditional healing practices on health outcomes in American Indian and Alaska Native communities

**Workshop Handouts and Materials**

1. Facilitator’s Guide
2. Computer and AV Technology
3. Evaluations

**Suggested Agenda and Timeline**

Suggested Total Time Frame: 60 minutes

1. An Introduction to Traditional Healing and American Indian and Alaska Native Health (15-20 minutes)
2. Delivery of Traditional Healing Services (7-8 minutes)
3. Traditional Healing Services: Policy Landscape and Successful Cases (15-20 minutes)
4. Open and Practice Discussions, Q+A (10 minutes)

**Slide Instructions**

*Please review module slides for up-to-date information and change, if appropriate.*

**Title Slide:**

| Update presenter information and presentation date. Introduce yourselves and encourage participant introductions if logistics allow. A land acknowledgement is welcome, depending on facilitator experience. |
| --- |

**Disclosures Slide:**

| Disclose personal and professional financial relationships or conflicts of interest that may bias your presentation, also stating that the module was adapted for use from MedEdPORTAL. |
| --- |

**Learning Objectives Slide:**

| Do not read learning objectives verbatim. We encourage facilitators to outline mutual goals for the presentation, encourage learners’ participation with facilitators, and to approach the content with open minds. |
| --- |

**Agenda 1:**

| In order to best understand health for American Indian and Alaska Native people and the relevance of traditional healing, it is important to have a foundation on which to build. In this section of the module, a general overview is provided to answer common questions regarding AI/AN people. |
| --- |

**Slide 5: A Primer on Language and General Principles**

| Language, and preferences for terminology, are constantly changing. Indigenous Peoples comprise 6 percent of the global population, but more than 15 percent of those experiencing severe poverty. In the US, there are residents Indigenous to North, Central, and South America. There are also Indigenous residents from other countries residing in the United States, but we do not readily track that information. The focus of this presentation is on American Indian and Alaska Native communities who have a specific “government-to-government” relationship with the United States. This relationship calls for the provision of culturally-relevant health care, education, and other social services to federally-recognized AI/AN Tribes and Villages in return for Indigenous land rights. The federal government does not recognize the autonomy of Native Hawaiian Nations. |
| --- |

**Slide 6: A Primer on Language and General Principles (Cont.)**

| Read the definition of American Indian and Alaska Native, explaining that this is an official racial identifier used by the United States. Some individuals and communities prefer Native American or Indigenous, or to be referred to by their specific tribal affiliations. |
| --- |

**Slide 7: American Indian and Alaska Native Cultures**

| Indigenous communities have been stewards of their languages, cultures, and traditions since time immemorial. There is tremendous heterogeneity from community to community. Settler colonialism and cultural genocide almost brought an end to these communities, but there has been a shift from genocide and assimilation to state and federal policies affirming the right to tribal self-determinatiomn and self-governance. This shift started in the 1970s with passage of the Indian Self-Determination and Educational Assistance Act, and later with the American Indian Religious Freedom Act. AIRFA promoted cultural revitalization, including language and traditional healing. |
| --- |

**Slide 8: Hiawatha Indian Insane Asylum**

| Pictured is one of many Indian Insane Asylums that were used to imprison individuals with disabilities, mental illness, cultural knowledge keepers, and more. We encourage facilitators to read the description provided by UC Berkeley slowly, allowing learners to understand the significance of these institutions and the role that they played in interrupting the transmission of Indigenous traditional knowledge and oral histories. |
| --- |

**Slide 9: Conceptualizing AI/AN Health**

| In medical education, we tend to focus on the physician domain of health and high-level interventions, such as the use of pharmaceuticals. A more holistic picture of health, especially from an Indigenous worldview, also captures spiritual, mental, and social domains of health that interplay with each other. This cultural construction of health helps us understand that AI/AN or Indigenous healing practices are rooted more in the psycho-social domains, rather than physical domains. These practices vary from community to community, but may involve prayer, song, dancing, and more. |
| --- |

**Slide 10: NIMHD Research Framework**

| This slide is meant to emphasize that there are many social and structural determinants of AI/AN health, ranging from biological and behavioral to those that are rooted in systems of oppression.  From the NIH: This health disparities framework emphasizes the genetic, biological, environmental, social, political, and cultural influences on the health of individuals and populations. It highlights multiple points of intersection and their iterative effects. All elements operate in the context of the birth status of people in the target population, such as genetics, race, ethnicity, age, sex, and tribal affiliation, as well as ascribed status deriving from education, income, and occupation.  It offers a systematic approach by which to identify and locate influences that may be especially relevant to understanding the health and well being of American Indians and Alaska Natives. This is particularly true in regard to determinants thought to be salient for this population, but which are often not considered by conventional research because they reside outside of the normal experience of non-Native groups.  Examples include spirituality, collective resilience, historical trauma, non-biomedical constructions of illness, traditional healing, boarding school education, alternate financing and structure of healthcare, tribal sovereignty, major federal Indian policies, and the like. |
| --- |

**Slide 11: Question 1 - What is “Traditional” healing?**

| Allow learners time and space to reflect on their own understanding of traditional healing. Encourage learners to share their perspectives or use a chat function (if in a virtual setting) to answer the question. Facilitators may also share personal examples depending on their backgrounds. |
| --- |

**Slide 12: AI/AN Traditional Healing Practices**

| Please read the definition of Indigenous traditional healing as defined by the World Health Organization. Place special emphasis on the phrasing “whether explicable or not,” which reminds learners that principles like cultural humility remind us that it is not necessary for non-Indigenous Peoples to fully understand how and why Indigenous healing practices are important to Indigenous Peoples. |
| --- |

**Slide 13: Examples of Traditional Healing**

| Traditional healing practices vary from community to community, and generalizations are very difficult to accurately make. In addition, some tribal nations may keep their practices private from those not from the community. However, it is important for learners to have concrete examples of what traditional healing may look like to a patient.  In order to have the most accurate picture of what traditional healing might look like in a specific patient population, learners may benefit from looking into the specific communities that they practice near. Some communities may share examples of their traditional practices with outsiders. |
| --- |

**Slide 14: Misconceptions with Traditional Healing**

| Indigenous cultures and practices have long been appropriated and disturbed. Traditional healing is not the same as naturopathy or naturopathic medicine. Further, these practices predate modern medical practice and concepts of statistical significance and experimental designs, making it unlikely that academic literature explores these topics with a Western lens. Unfortunately, physicians and the medical community have not always appreciated the importance of traditional healing and traditional healers from Indigenous communities, as shown in the caption from the American Medical Association’s Strategic Plan for Health Equity. |
| --- |

**Slide 15: Question 2 - Can you think of other non-Western or spiritually-directed services that are already provided in healthcare systems for patients from other racial and ethnic groups?**

| Allow learners time and space to reflect on their own understanding of traditional healing. Encourage learners to unmute or speak up, if able, or to use chat functions to answer the question if comfortable. Facilitators may also share personal examples depending on their backgrounds. In previous sessions, participants commonly share figures and practices such as Shamans, Imams, acupuncture, and meditation. |
| --- |

**Slide 16: Culture as Prevention and Traditional Healing Practices**

| Cultural identification to an Indigenous person’s specific culture can improve health intervention outcomes and improve mental health and wellness. A number of studies, particularly in the behavioral health realm have seen this, even warranting recognition and promotion from the U.S. Substance Abuse and Mental Health Services Administration. |
| --- |

**Slide 17: Question 3: Why is traditional healing important for AI/AN patients?**

| Allow learners time and space to reflect on their own understanding of traditional healing. Encourage learners to unmute or speak up, if able, or to use chat functions to answer the question if comfortable. Facilitators may also share personal examples depending on their backgrounds. |
| --- |

**Slide 18: Video Example**

| This slide is intended to serve as a video example of traditional healing and its impact on patients and outcomes. The authors recommend a 3-5 minute video including a patient’s experience or a traditional healer’s perspective on the importance of traditional healing. Examples include:   1. Guy’s Story: Indigenous Traditional Medicine in a Cancer Story (<https://www.youtube.com/watch?v=asJ7pWaByGc>) 2. Dr. Melissa Lewis: Bringing Cultural Ways of Healing to Modern Medicine (<https://www.youtube.com/watch?v=dJKo5MTk8SQ>) 3. I am an Alaska Native Healer \| Indie Alaska   (https://www.youtube.com/watch?v=MyOIw78pwCA) |
| --- |

**Slide 19 Video Debrief**

| Ask the audience if there are any questions or comments about the video before advancing to an overview of the Indian Health Service, a federal agency within the U.S. Department of Health and Human Services. This is a great opportunity to solicit reaction to the practices highlighted in the video and if it helped participants understand why traditional healing is important to AI/AN patients. |
| --- |

**Slide 20: Question 4 - Why do American Indian and Alaska Native persons have a right to health care?**

| Allow learners time and space to reflect on their own understanding of this question. Encourage learners to unmute or speak up, if able, or to use chat functions to answer the question if comfortable. |
| --- |

**Slide 21: Background - Historical Context of the Right to Health Care**

| The authority to provide AI/AN health care stems from the Constitution, which states that treaties are the supreme law of the land. Treaties with AI/AN tribes, coupled with judicial rulings, executive orders, and other agreements have led to a “trust responsibility,” wherein the U.S. is responsible for providing comprehensive health care services to tribal members. This is based on their membership in federally-recognized tribes, not because of their racial identification as AI/AN. |
| --- |

**Slide 22: The Indian Health Service - A Timeline**

| The IHS is now housed in the Department of Health and Human Services, but funded by the Interior Appropriations Bill. Historically, the IHS, formerly the Office of Indian Affairs, was housed in the Department of War. The agency was responsible for containing the spread of prevalent diseases in Indigenous communities (usually on reservations) away from non-Indigenous communities. |
| --- |

**Slide 23: The Indian Health Service**

| The IHS started to promote AI/AN health and wellness in the 1950s. While underfunded, it oversees a health care delivery system for nearly 3 million tribal members in 37 states. We encourage facilitators to read the agency’s mission and vision statements. |
| --- |

**Slide 24: IHS Regions and Service Areas**

| There are 12 IHS Service Areas. Each Service Area or Region coordinates care at hospitals, clinics, school satelites, and related facilities. There is 1 Region dedicated exclusively to the Navajo Nation. Each Region is appropriated funds to design health care services as they see fit for their patients. |
| --- |

**Slide 25: Tribal Self-Governance**

| Read: “Tribal leaders and members are in the best position to understand the health care needs and priorities of their communities.”  This can include coordination of care with traditional healers and designing health facilities with this intention. |
| --- |

**Slide 26: AI/AN Health Care Delivery Today**

| Today, most IHS facilities are directly operated by Tribes. Other facilities are operated by the federal government or as Urban Indian organizations (federally-qualified health centers). There are no IHS hospitals in California, where the majority of facilities are ambulatory clinics operated by tribes. A facility run by the government can transition control to tribal leads if a request is made. Importantly, while more than 75 percent of AI/ANs live in urban areas, only 1 percent of the IHS budget is dedicated to urban AI/AN health care. The Urban Indian Health Institute in Seattle, WA has a successful traditional medicine program that they fund with their own monies. Also take time to engage trainees with the Discussion question. Ask them to take a few minutes to try and find out and report back. |
| --- |

**Slide 27: Agenda 3**

| Ask the audience if there are any questions or comments before advancing to Traditional Healing Services for an overview of the current policy landscape, including IHS and insurance coverage, as well as examples of successful traditional healing clinics. |
| --- |

**Slide 28: Supporting Practice for Traditional Healing Services**

| Read quote from Minnesota Department of Human Services: “Research consistently points to the value of traditional healing practices designed and delivered by American Indians, for American Indians. Traditional healing for American Indians has outcomes equivalent to conventional interventions in other populations.”  In 2022, the White House Office of Science and Technology Policy published government-wide guidance recognizing and including Indigenous Knowledge in federal research, policy, and decision-making.  This guidance calls attention to the principle that Indigenous Knowledge and other forms of knowledge, such as Western methods of scientific inquiry, do not depend on each other for validation, and each system can support, rather than work against each other. |
| --- |

**Slide 29: Question 5 - What are some of the barriers to accessing traditional healing for AI/AN patients?**

| Allow learners time and space to determine potential barriers to traditional healing. Encourage learners to unmute or speak up, if able, or to use chat functions (if virtual) to answer the question if comfortable. Facilitators may also share personal examples depending on their backgrounds. |
| --- |

**Slide 30: Barriers to Implementing Traditional Healing Services (THS)**

| Patients may be prevented from accessing THS due to limitations from the Indian Health Service and insurance payors. The Indian Health Service does not authorize payment or reimbursement for THS, and few insurance plans include THS. One avenue for coverage is through Section 1115 demonstration waivers, such as those in Arizona, California, and New Mexico, which allows Medicare and Medicaid services to cover these costs. These waivers require a framework for THS delivery, including approved services, care agreements between physicians and traditional healers, and other general principles such as consultation before ingestion or inhalation and a defined evaluation plan. |
| --- |

**Slide 31: Considerations for Your Practice on Incorporating Traditional Healers into Patient Care**

| Physicians should actively invite patients to share their experiences and priorities in regards to their health. They can play an active role in incorporating traditional healers into the health care setting by designing intake forms as well as adapting the patient interview. This slide can be made more interactive by inviting the audience to discuss alternative ways to incorporate traditional healing into the patient interview. |
| --- |

**Slide 32: Successful Cases: Southcentral Foundation Traditional Healing Clinic**

| The Southcentral Foundation is a tribally-run health center located in Anchorage, Alaska. Customer-owners, the equivalent of patients in other health systems, may be seen at Southcentral’s Traditional Healing Clinic with a referral from their primary care provider or other medical specialty. Patients are offered an array of services, including physical treatments, counseling sessions, and cultural classes meant to foster connection to Alaska Native culture. In order to quantify the importance of this service to customer-owners, The Foundation administered a survey in 2016. 89% of customer-owners stated that the services provided at this clinic were either an “extremely important” or an “important” aspect of their health care, demonstrating the overwhelming majority who felt this is an important service in their health. |
| --- |

**Slide 33: Winslow Indian Health Care Center Navajo Traditional Medicine Program (TMP)**

| Alternatively, the Winslow Indian Health Care Center Navajo Traditional Medicine Program is a program located in a federally-run IHS facility. Opening in 2019, it incorporates traditional healing methods to promote health and wellness while restoring balance and harmony. The program also offers a training program for future Navajo cultural leaders, resulting in certification to ensure that those providing traditional healing services are recognized. |
| --- |

**Slide 34: Considerations for Care of American Indian and Alaska Native Patients in the Clinic**

| Reaffirm the critical aspects of caring for AI/AN populations. As with all patients, shared-decision making is a crucial part of creating and maintaining an effective patient-physician relationship in AI/AN populations. Physicians should affirm the worldviews of Indigenous patients, and be aware of practices important to the patient and their health, including cultural and spiritual values. Physicians should also consider that patients may not readily share information on these subjects due to historical and contemporary stigma and discrimination, and may also receive traditional healing services without informing their physician-led healthcare team. |
| --- |

**Slide 35: Open Discussion**

| Encourage learners to voice concerns and questions with regards to incorporating traditional healing, and challenge learners to use their knowledge to describe the role of a traditional healer in the physician-led healthcare team. Some examples of concerns may include forgoing physician care to see a traditional healer or not disclosing relevant health information to the health care team. |
| --- |

**Slide 36: Practice Discussion**

| Learners can either divide into small groups or self-test to assess their knowledge and ability to follow the given prompt, depending on remaining time. This scenario applies knowledge and learning points previously discussed in the PowerPoint. It also allows them to reflect on the material and how they might change their own practice in the future. It is also useful here to offer that a key barrier to implementation may be a lack of understanding of cultural protocols in communicating and requesting traditional healer assistance and/or teachings. Healthcare teams should ask with humility what these protocols may be and how they can respect the patients’ cultural norms and knowledge safeguards. |
| --- |

**Slide 37: Additional Resources**

| We encourage facilitators to provide learners with additional resources to further their own education and to complete the post-evaluation. |
| --- |

**Slide 38: Concluding Slide**

| Update facilitator contact information and organizational sponsors. Thank the learners for their time and attention to this module. |
| --- |

**References**

1. Reshaping the Journey: American Indians and Alaska Natives in Medicine. *Association of American Medical Colleges*. Published October 31, 2018. Accessed December 5, 2023. <https://store.aamc.org/reshaping-the-journey-american-indians-and-alaska-natives-in-medicine.html>
2. Sánchez-Rivera, A., Jacobs, P., Spence, C. A Look at the Largest American Indian and Alaska Native Tribes and Villages in the Nation, Tribal Areas and States. *United States Census Bureau*. Published October 3, 2023. Accessed December 5, 2023. <https://www.census.gov/library/stories/2023/10/2020-census-dhc-a-aian-population.html>
3. About IHS. *Indian Health Service*. Accessed December 5, 2023. <https://www.ihs.gov/aboutihs/>
4. Manson, S. NIMHD Minority Health and Health Disparities Research Framework: Adapted to reflect historical and socio-cultural influences for American Indian and Alaska Native Nations. *National Institute on Minority Health and Health Disparities*.Published 2017. Accessed December 5, 2023. <https://www.nimhd.nih.gov/about/overview/research-framework/adaptation-framework.html>
5. Pacheco CM, Daley SM, Brown T, Filippi M, Greiner KA, Daley CM. Moving forward: breaking the cycle of mistrust between American Indians and researchers. *Am J Public Health*. 2013;103(12):2152-2159. doi:10.2105/AJPH.2013.301480
6. Kahn CB, James D, George S, et al. Diné (Navajo) Traditional Knowledge Holders' Perspective of COVID-19. *Int J Environ Res Public Health*. 2023;20(4):3728. Published 2023 Feb 20. doi:10.3390/ijerph20043728
7. Redvers N, Blondin B. Traditional Indigenous medicine in North America: A scoping review. *PLoS One*. 2020;15(8):e0237531. Published 2020 Aug 13. doi:10.1371/journal.pone.0237531
8. Prabhakar, A., Mallory, B. Guidance for Federal Departments and Agencies on Indigenous Knowledge. *White House Office of Science and Technology Policy*. Published November 30, 2022. Accessed December 5, 2023. <https://www.whitehouse.gov/wp-content/uploads/2022/12/OSTP-CEQ-IK-Guidance.pdf>
9. Garcia, A., Castro, M., Sanchez, JP. Social and Structural Determinants of Urban American Indian and Alaska Native Health: A Case Study in Los Angeles. *MedEdPORTAL*. Published January 1, 2019. https://doi.org/10.15766/mep_2374-8265.10825.
10. Tobey, M., Sacks, C., Foster, D., Norman, D., Karol, S., Lee, P. American Indian Health in South Dakota: A Health Systems Case. *MedEdPORTAL*. Published January 1, 2014. https://doi.org/10.15766/mep_2374-8265.9869
11. Jaramillo ET, Sommerfeld DH, Haozous EA, Brunner A, Willging CE. Causes and Consequences of Not Having a Personal Healthcare Provider Among American Indian Elders: A Mixed-Method Study. *Front Public Health*. Published March 2, 2022. doi:10.3389/fpubh.2022.832626
12. Invisible Tribes: Urban Indians and Their Health in a Changing World. *Urban Indian Health Commission*. Published January 1, 2007. Accessed December 5, 2023. <https://www2.census.gov/cac/nac/meetings/2015-10-13/invisible-tribes.pdf>
13. Allice, I., Acai, A., Ferdossifard, A., Wekerle, C., Kimber, M. Indigenous Cultural Safety in Recognizing and Responding to Family Violence: A Systematic Scoping Review. *International Journal of Environmental Research and Public Health*. 2022; 19(24):16967. https://doi.org/10.3390/ijerph192416967
14. MacLean, TL, Qiang, JR, Henderson, L., Bowra, A., Howard, L., Pringle, V., Butsang, T., Rice, E., Di Ruggiero E., Mashford-Pringle A. Indigenous Cultural Safety Training for Applied Health, Social Work, and Education Professionals: A PRISMA Scoping Review. *International Journal of Environmental Research and Public Health*. 2023; 20(6):5217. https://doi.org/10.3390/ijerph20065217
15. Gampa V, Bernard K, Oldani MJ. Racialization as a Barrier to Achieving Health Equity for Native Americans. *AMA J Ethics*. 2020;22(10):E874-E881. Published 2020 Oct 1. doi:10.1001/amajethics.2020.874
16. Chowkwanyun M, Reed AL Jr. Racial Health Disparities and Covid-19 - Caution and Context. *N Engl J Med*. 2020;383(3):201-203. doi:10.1056/NEJMp2012910
17. Gampa V, Bernard K, Oldani MJ. Racialization as a Barrier to Achieving Health Equity for Native Americans. *AMA J Ethics*. 2020;22(10):E874-E881. Published 2020 Oct 1. doi:10.1001/amajethics.2020.874
18. Zestcott CA, Spece, L., McDermott D., Stone J. Health Care Providers' Negative Implicit Attitudes and Stereotypes of American Indians. *J Racial Ethn Health Disparities*. 2021;8(1):230-236. doi:10.1007/s40615-020-00776-w
19. Henson M, Sabo S, Trujillo A, Teufel-Shone N. Identifying Protective Factors to Promote Health in American Indian and Alaska Native Adolescents: A Literature Review. *J Prim Prev*. 2017;38(1-2):5-26. doi:10.1007/s10935-016-0455-2
20. Brockie TN, Campbell JC, Dana-Sacco G, et al. Cultural Protection from Polysubstance Use Among Native American Adolescents and Young Adults. *Prev Sci*. 2022;23(7):1287-1298. doi:10.1007/s11121-022-01373-5
21. Venner KL, Donovan DM, Campbell ANC, et al. Future directions for medication assisted treatment for opioid use disorder with American Indian/Alaska Natives. *Addict Behav*. 2018;86:111-117. doi:10.1016/j.addbeh.2018.05.017

# Arizona’s Section 1115 Waiver Renewal Request (2022-2027). *Arizona Health Care Cost Containment System*. Published December 22, 2020. Accessed December 5, 2023. <https://www.azahcccs.gov/Resources/Federal/waiverrenewalrequest.html>

1. Tribal and Indian Health Program Representatives Meeting. *California Department of Health Care Services*. Published February 24, 2022. Accessed December 5, 2023. <https://www.dhcs.ca.gov/Documents/Tribes-and-Indian-Health-Program-Representatives-Meeting-Presentation-02-24-22.pdf>
