# Supplementary material for: An Introduction to Traditional Healing in American Indian and Alaska Native Communities
Source: MedEdPORTAL. 2025 Mar 7;21:11506. doi: 10.15766/mep_2374-8265.11506 (PMC11885593; doi:10.15766/mep_2374-8265.11506)
Supplement: Supplementary file 1 — Facilitator Guide.docxInstructional Slides.pptxTrainee Presurvey.docxTrainee Postsurvey.docx [file mep_2374-8265.11506-s001.zip › B. Instructional Slides.pptx]

## Slide 1
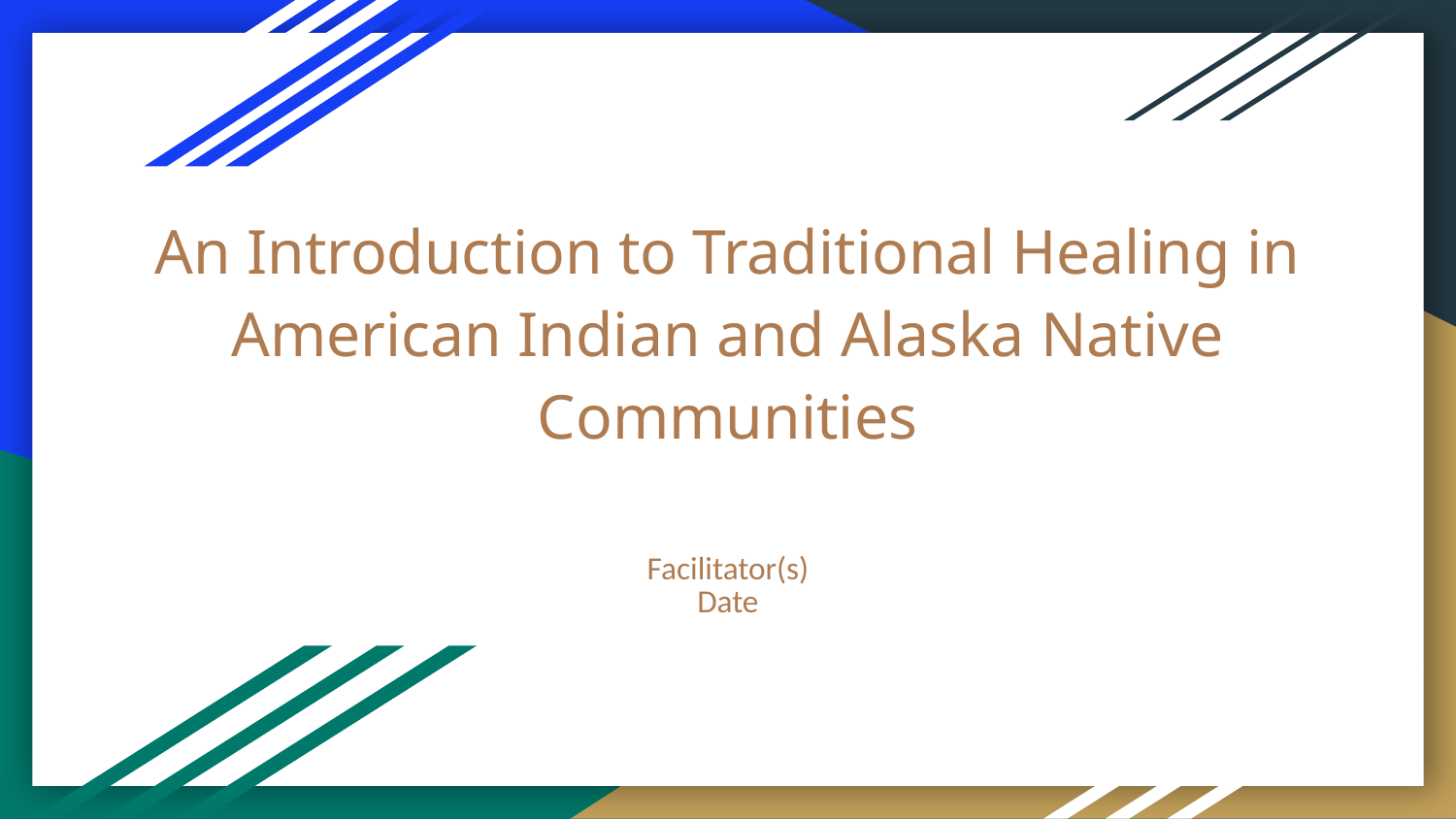

# An Introduction to Traditional Healing in American Indian and Alaska Native Communities
Facilitator(s)
Date

## Slide 2
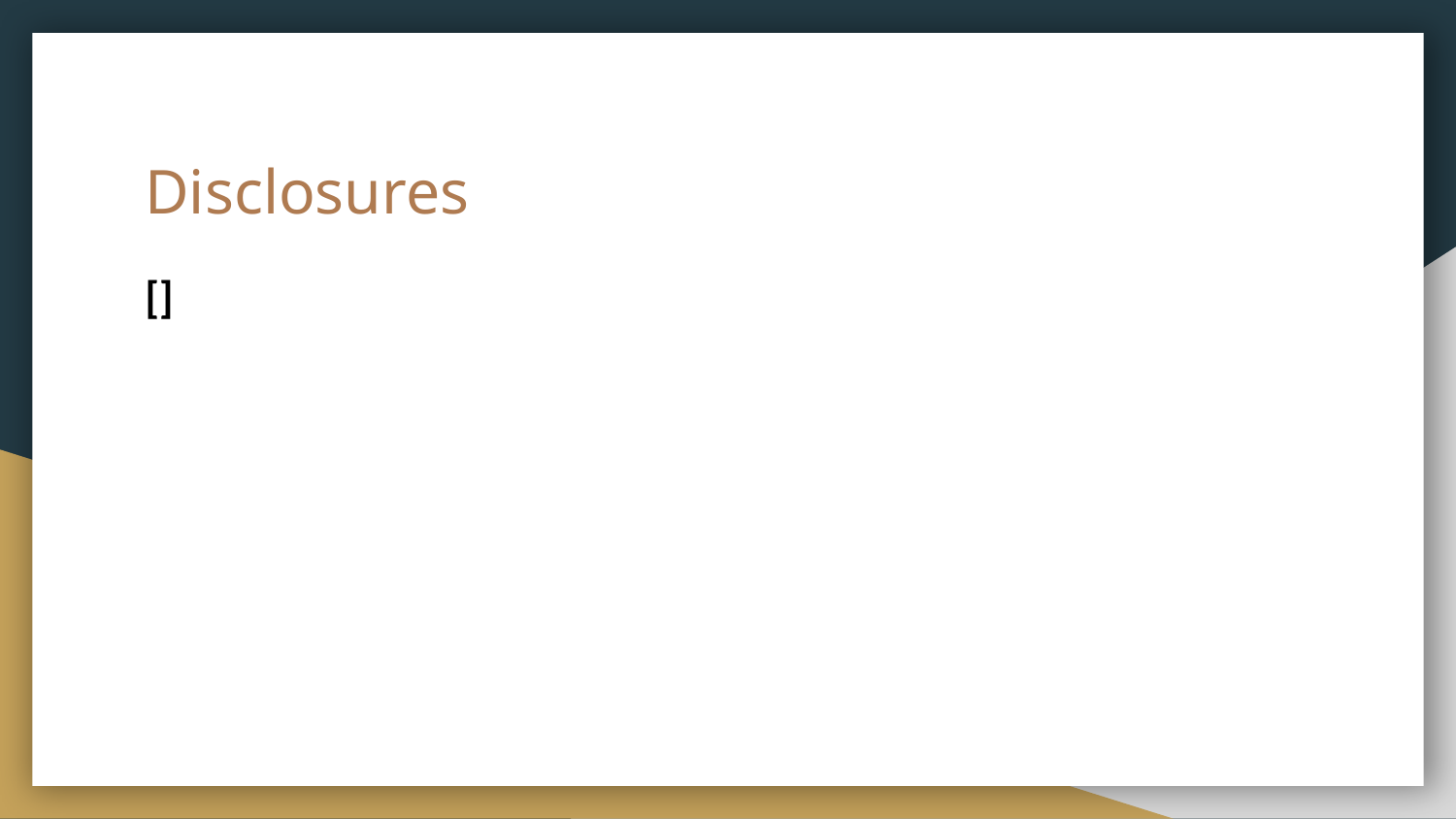

# Disclosures
[]

## Slide 3
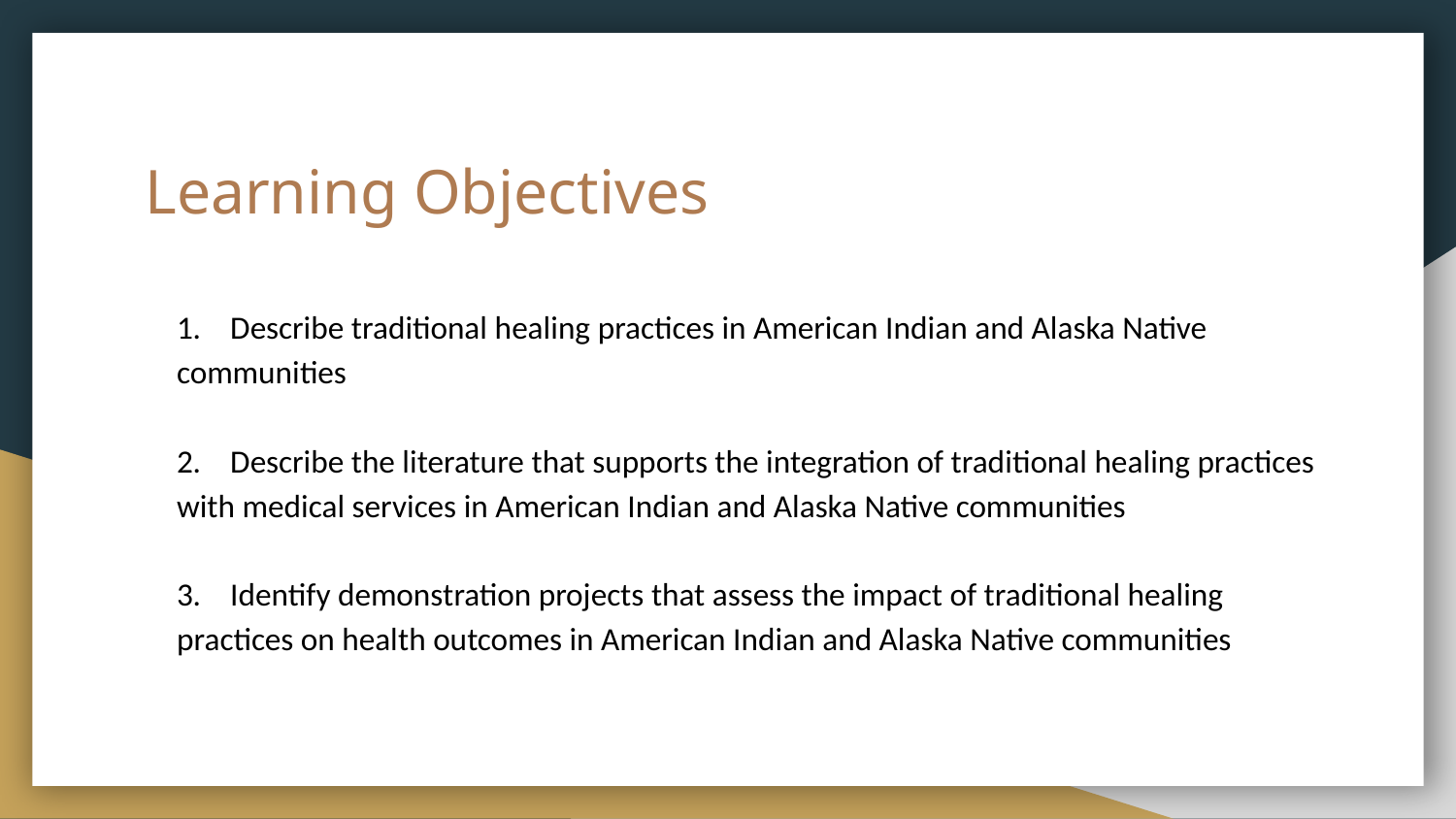

# Learning Objectives
1. Describe traditional healing practices in American Indian and Alaska Native communities
2. Describe the literature that supports the integration of traditional healing practices with medical services in American Indian and Alaska Native communities
3. Identify demonstration projects that assess the impact of traditional healing practices on health outcomes in American Indian and Alaska Native communities

## Slide 4
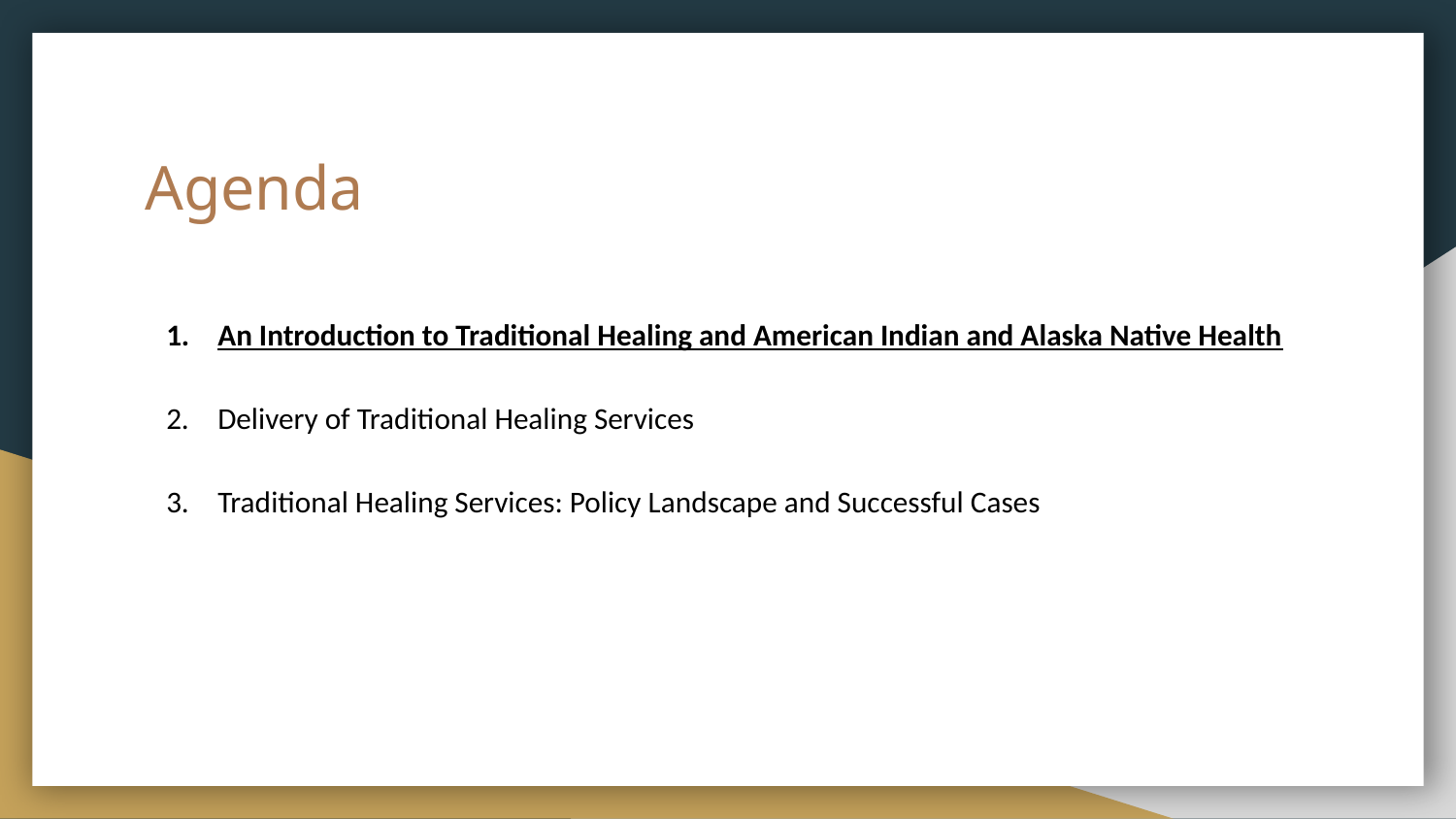

# Agenda
An Introduction to Traditional Healing and American Indian and Alaska Native Health
Delivery of Traditional Healing Services
Traditional Healing Services: Policy Landscape and Successful Cases

## Slide 5
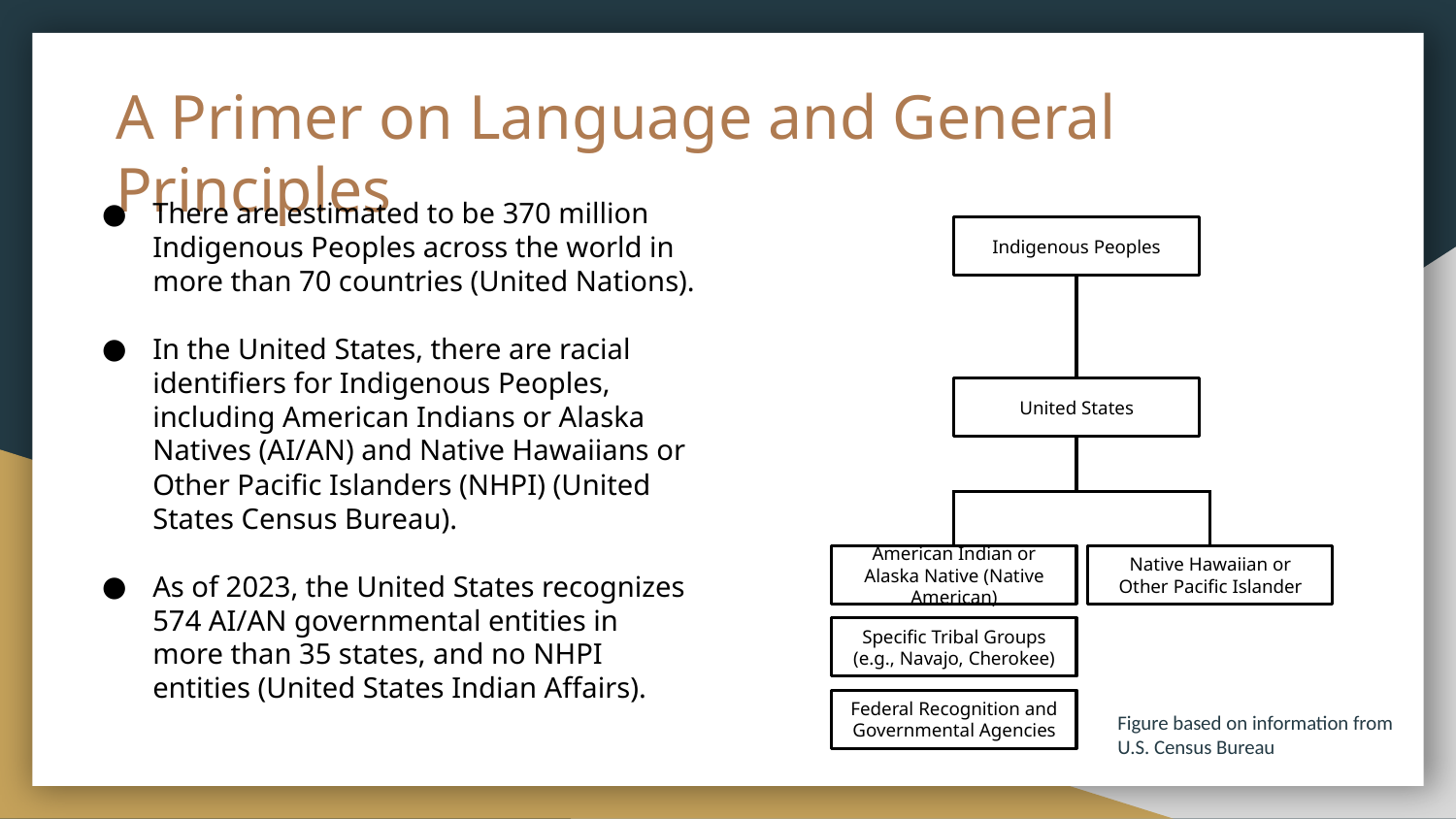

# A Primer on Language and General Principles
There are estimated to be 370 million Indigenous Peoples across the world in more than 70 countries (United Nations).
In the United States, there are racial identifiers for Indigenous Peoples, including American Indians or Alaska Natives (AI/AN) and Native Hawaiians or Other Pacific Islanders (NHPI) (United States Census Bureau).
As of 2023, the United States recognizes 574 AI/AN governmental entities in more than 35 states, and no NHPI entities (United States Indian Affairs).
Indigenous Peoples
United States
American Indian or Alaska Native (Native American)
Native Hawaiian or Other Pacific Islander
Specific Tribal Groups (e.g., Navajo, Cherokee)
Federal Recognition and Governmental Agencies
Figure based on information from U.S. Census Bureau

## Slide 6
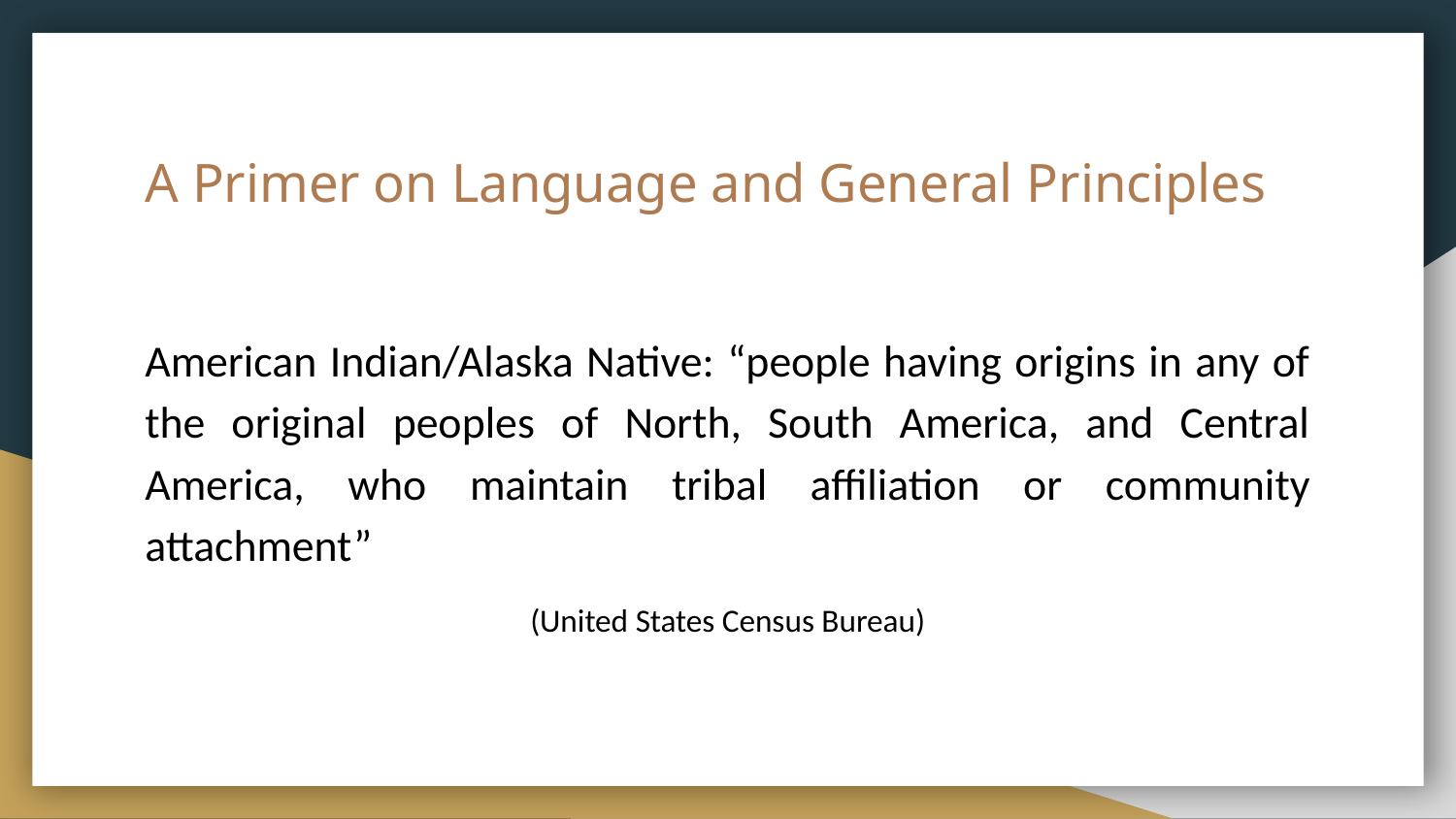

# A Primer on Language and General Principles
American Indian/Alaska Native: “people having origins in any of the original peoples of North, South America, and Central America, who maintain tribal affiliation or community attachment”
(United States Census Bureau)

## Slide 7
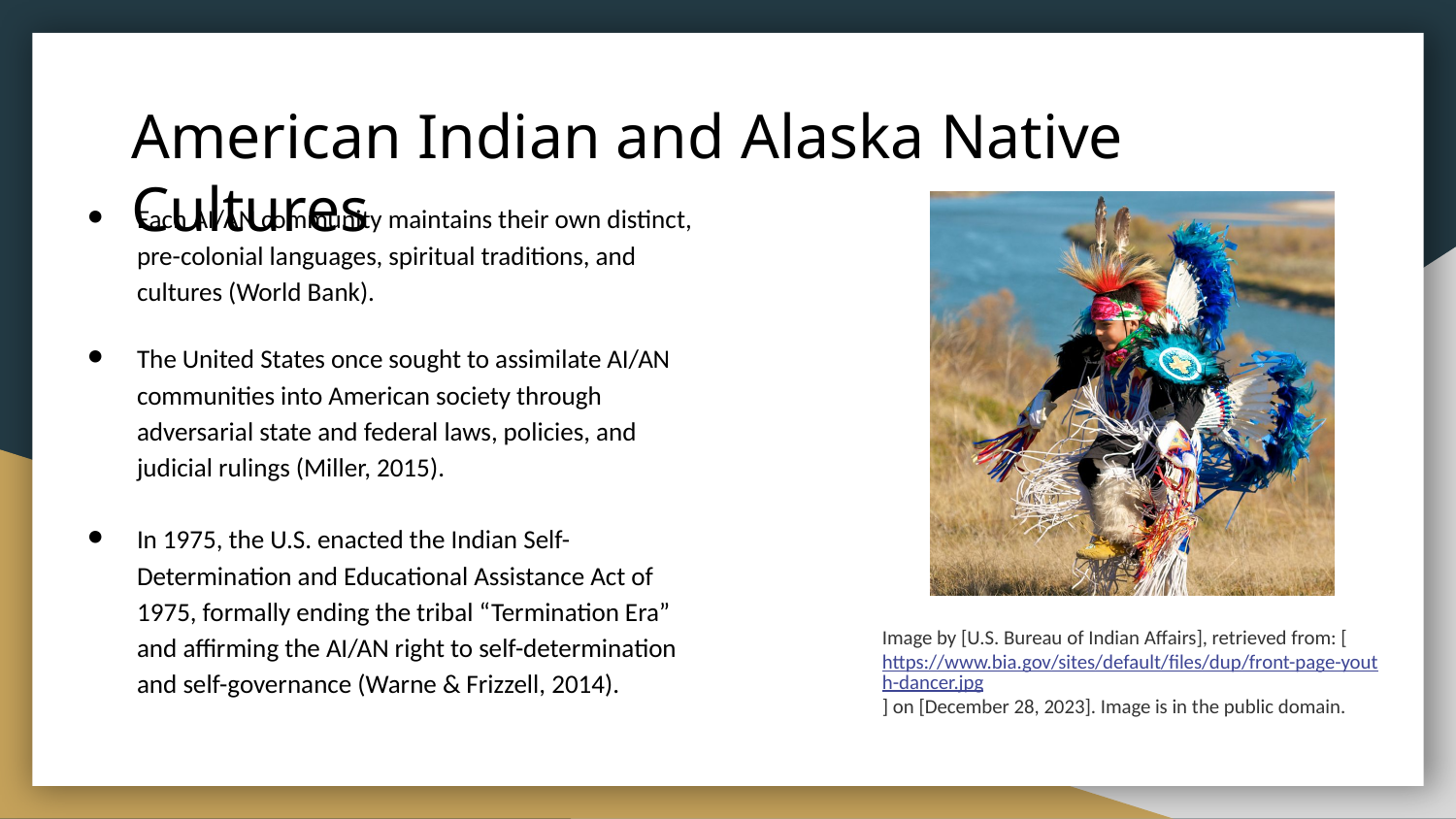

# American Indian and Alaska Native Cultures
Each AI/AN community maintains their own distinct, pre-colonial languages, spiritual traditions, and cultures (World Bank).
The United States once sought to assimilate AI/AN communities into American society through adversarial state and federal laws, policies, and judicial rulings (Miller, 2015).
In 1975, the U.S. enacted the Indian Self-Determination and Educational Assistance Act of 1975, formally ending the tribal “Termination Era” and affirming the AI/AN right to self-determination and self-governance (Warne & Frizzell, 2014).
Image by [U.S. Bureau of Indian Affairs], retrieved from: [https://www.bia.gov/sites/default/files/dup/front-page-youth-dancer.jpg] on [December 28, 2023]. Image is in the public domain.

## Slide 8
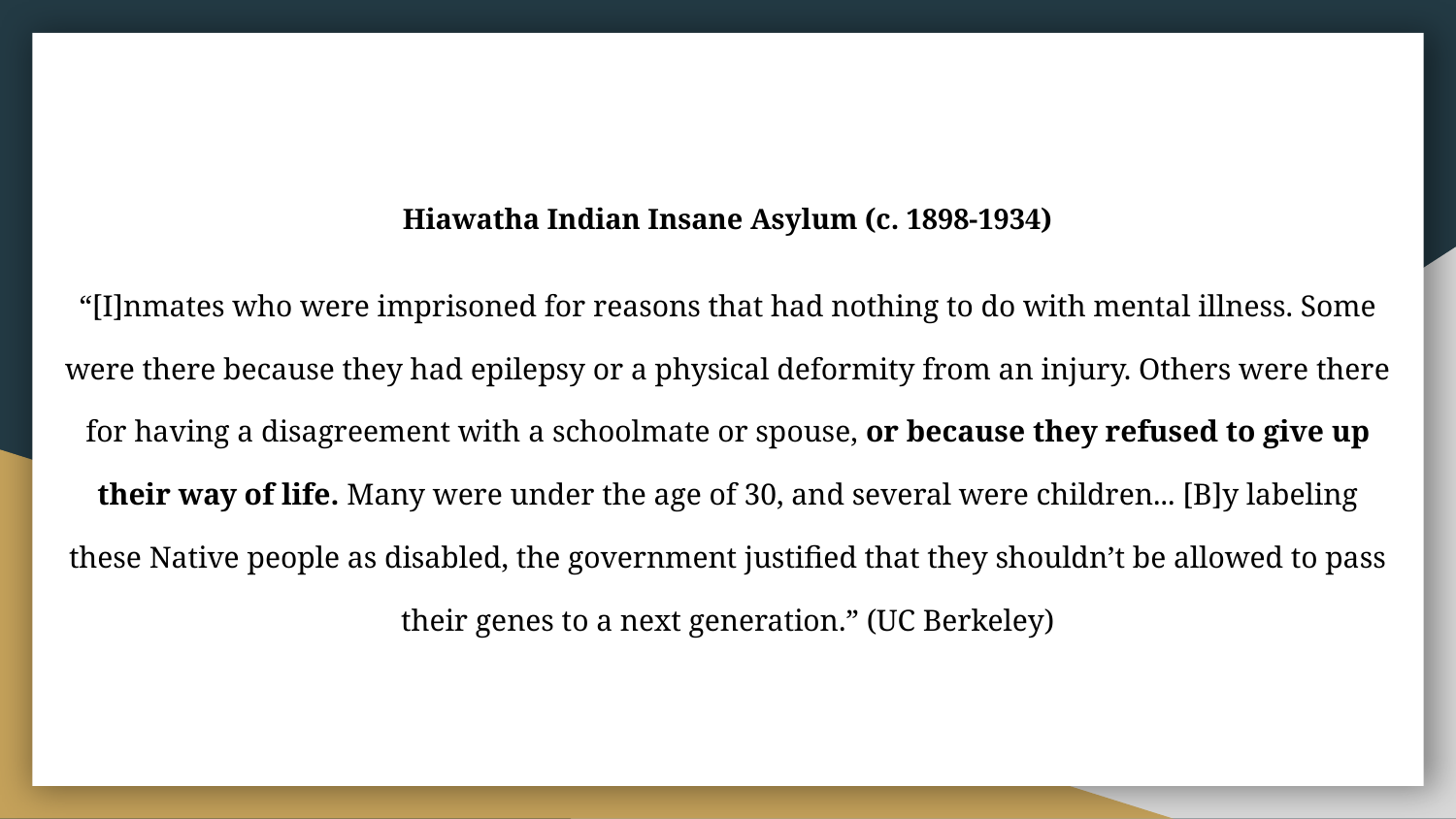

Hiawatha Indian Insane Asylum (c. 1898-1934)
“[I]nmates who were imprisoned for reasons that had nothing to do with mental illness. Some were there because they had epilepsy or a physical deformity from an injury. Others were there for having a disagreement with a schoolmate or spouse, or because they refused to give up their way of life. Many were under the age of 30, and several were children... [B]y labeling these Native people as disabled, the government justified that they shouldn’t be allowed to pass their genes to a next generation.” (UC Berkeley)

## Slide 9
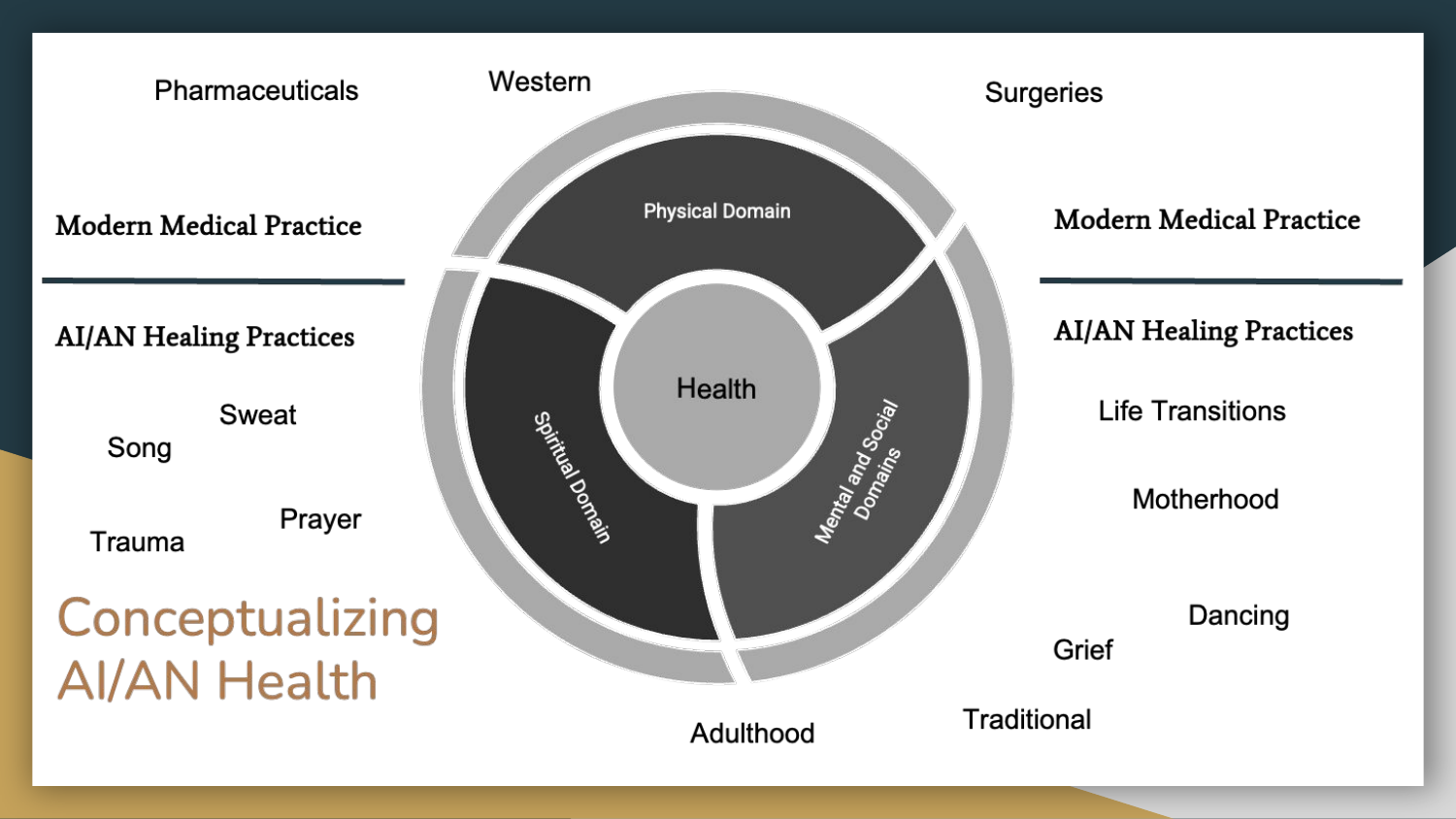

## Slide 10
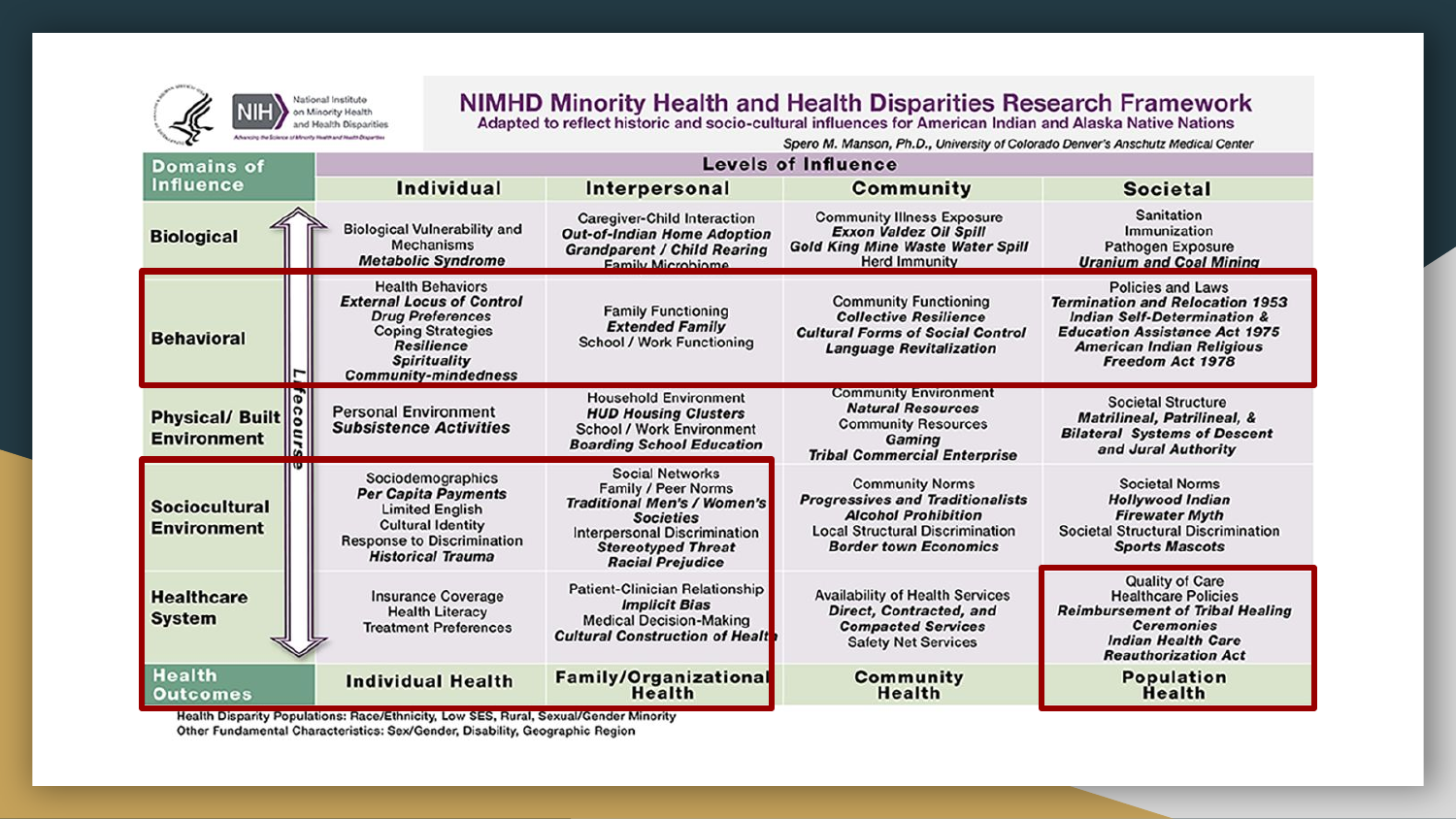

## Slide 11
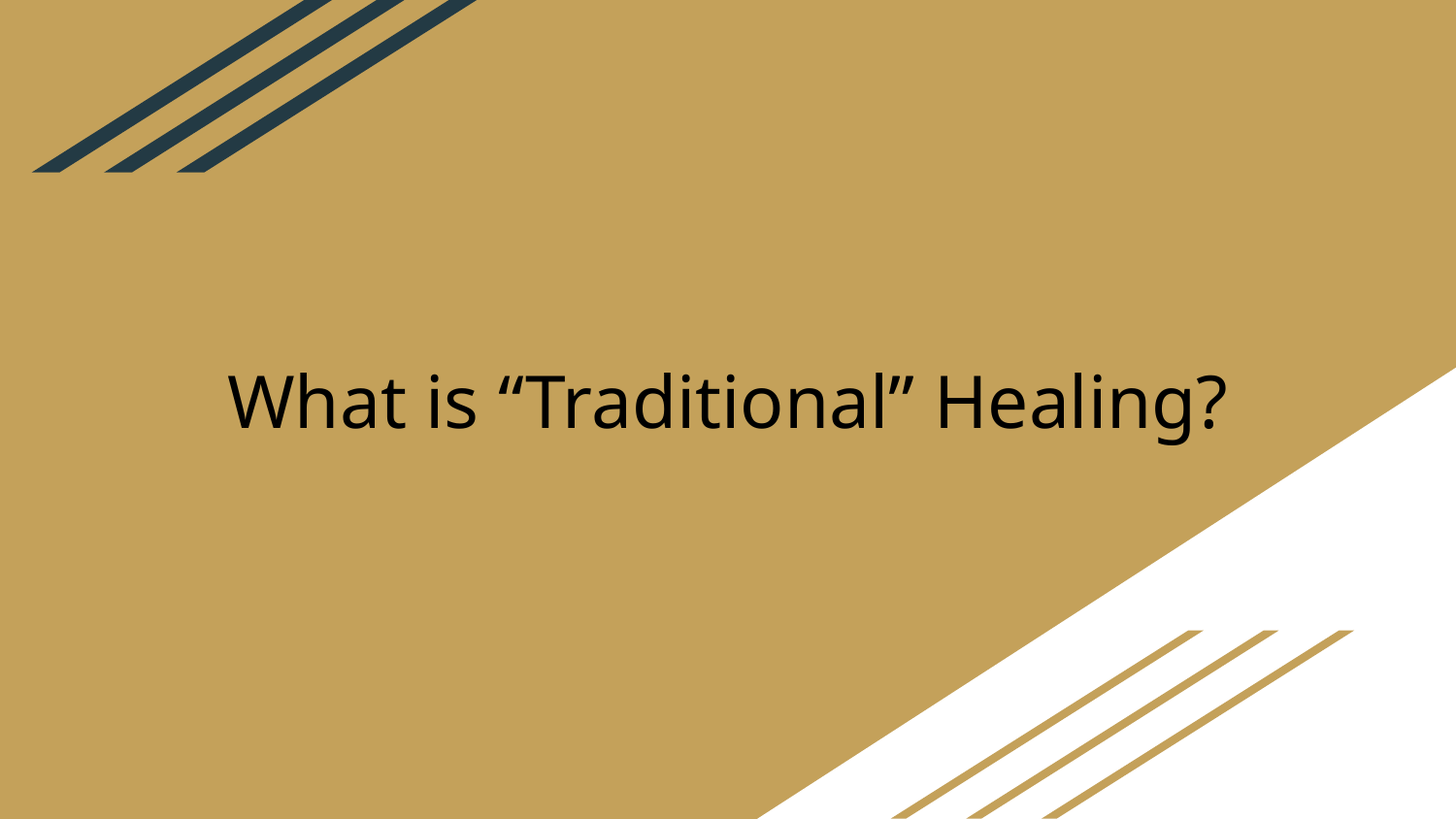

# What is “Traditional” Healing?

## Slide 12
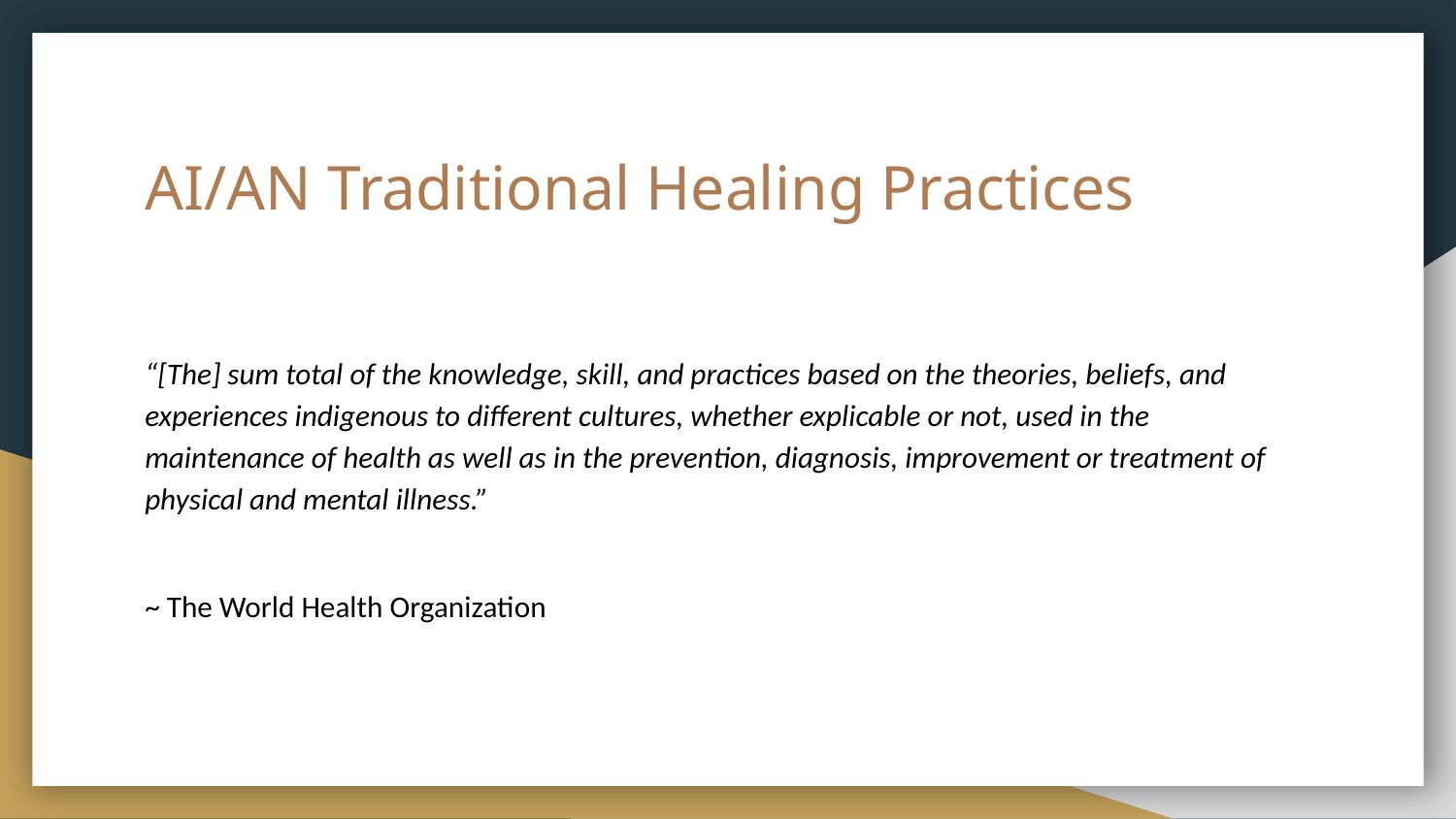

# AI/AN Traditional Healing Practices
“[The] sum total of the knowledge, skill, and practices based on the theories, beliefs, and experiences indigenous to different cultures, whether explicable or not, used in the maintenance of health as well as in the prevention, diagnosis, improvement or treatment of physical and mental illness.”
								~ The World Health Organization

## Slide 13
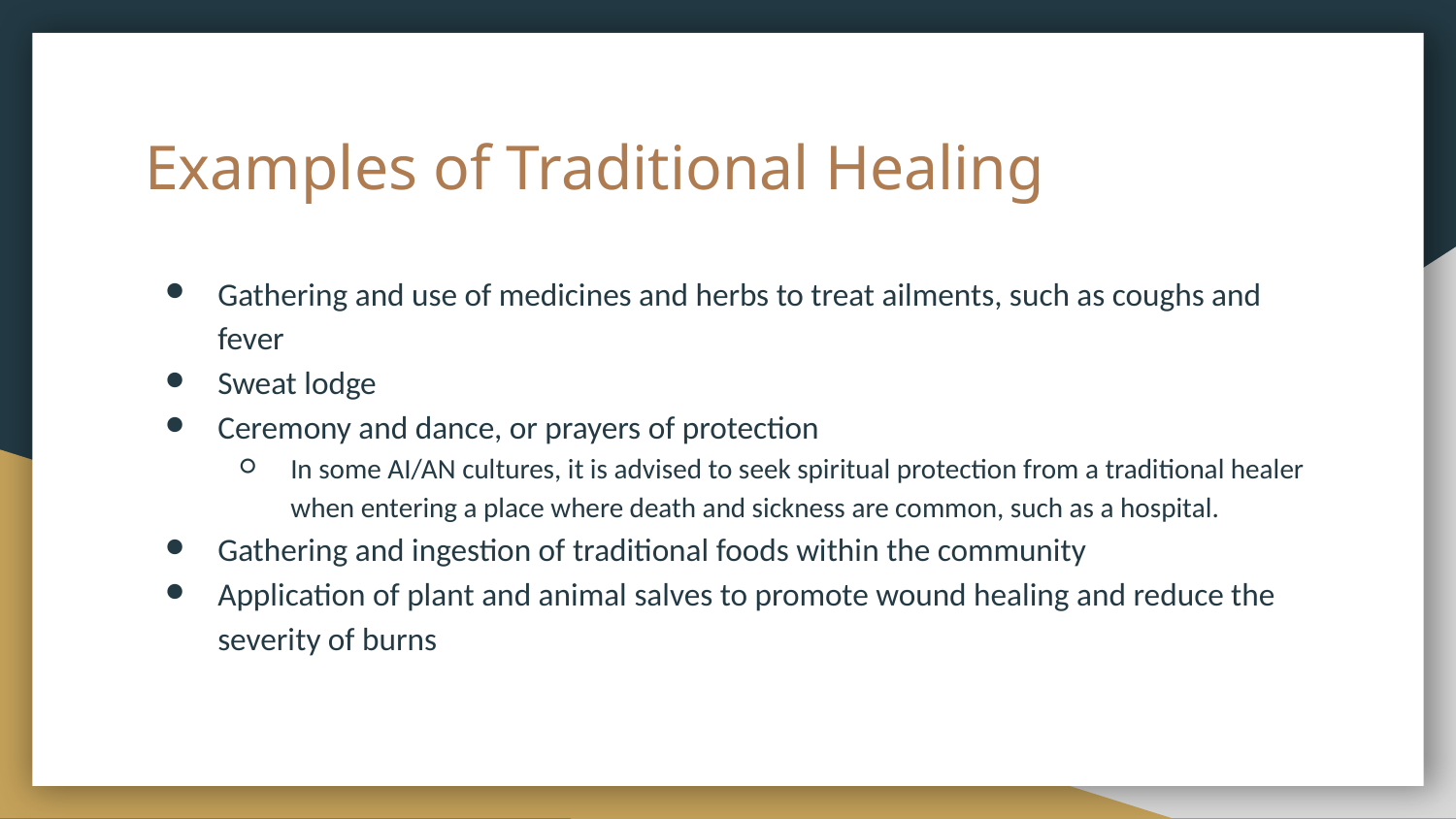

# Examples of Traditional Healing
Gathering and use of medicines and herbs to treat ailments, such as coughs and fever
Sweat lodge
Ceremony and dance, or prayers of protection
In some AI/AN cultures, it is advised to seek spiritual protection from a traditional healer when entering a place where death and sickness are common, such as a hospital.
Gathering and ingestion of traditional foods within the community
Application of plant and animal salves to promote wound healing and reduce the severity of burns

## Slide 14
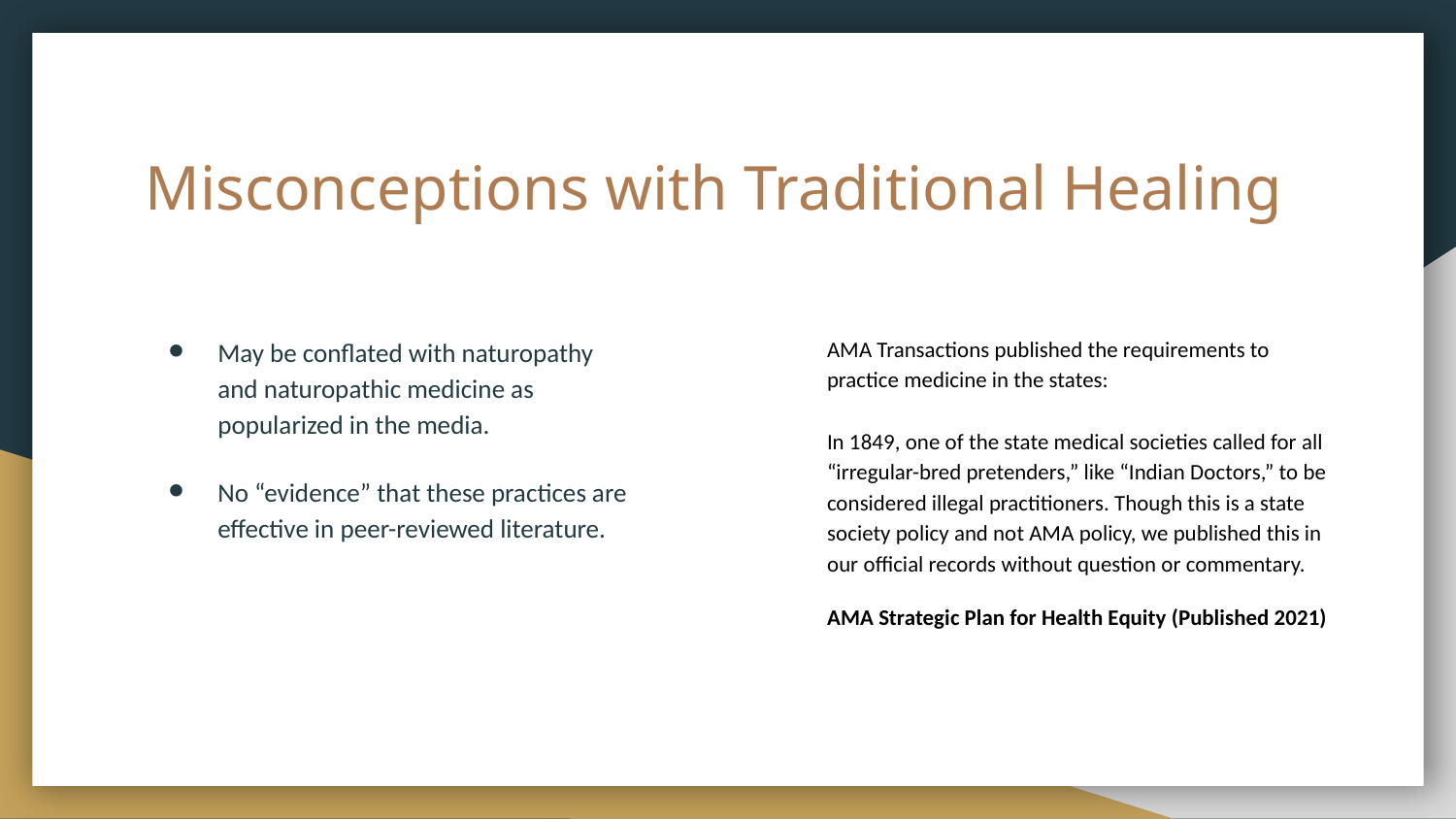

# Misconceptions with Traditional Healing
May be conflated with naturopathy and naturopathic medicine as popularized in the media.
No “evidence” that these practices are effective in peer-reviewed literature.
AMA Transactions published the requirements to practice medicine in the states:
In 1849, one of the state medical societies called for all “irregular-bred pretenders,” like “Indian Doctors,” to be considered illegal practitioners. Though this is a state society policy and not AMA policy, we published this in our official records without question or commentary.
AMA Strategic Plan for Health Equity (Published 2021)

## Slide 15
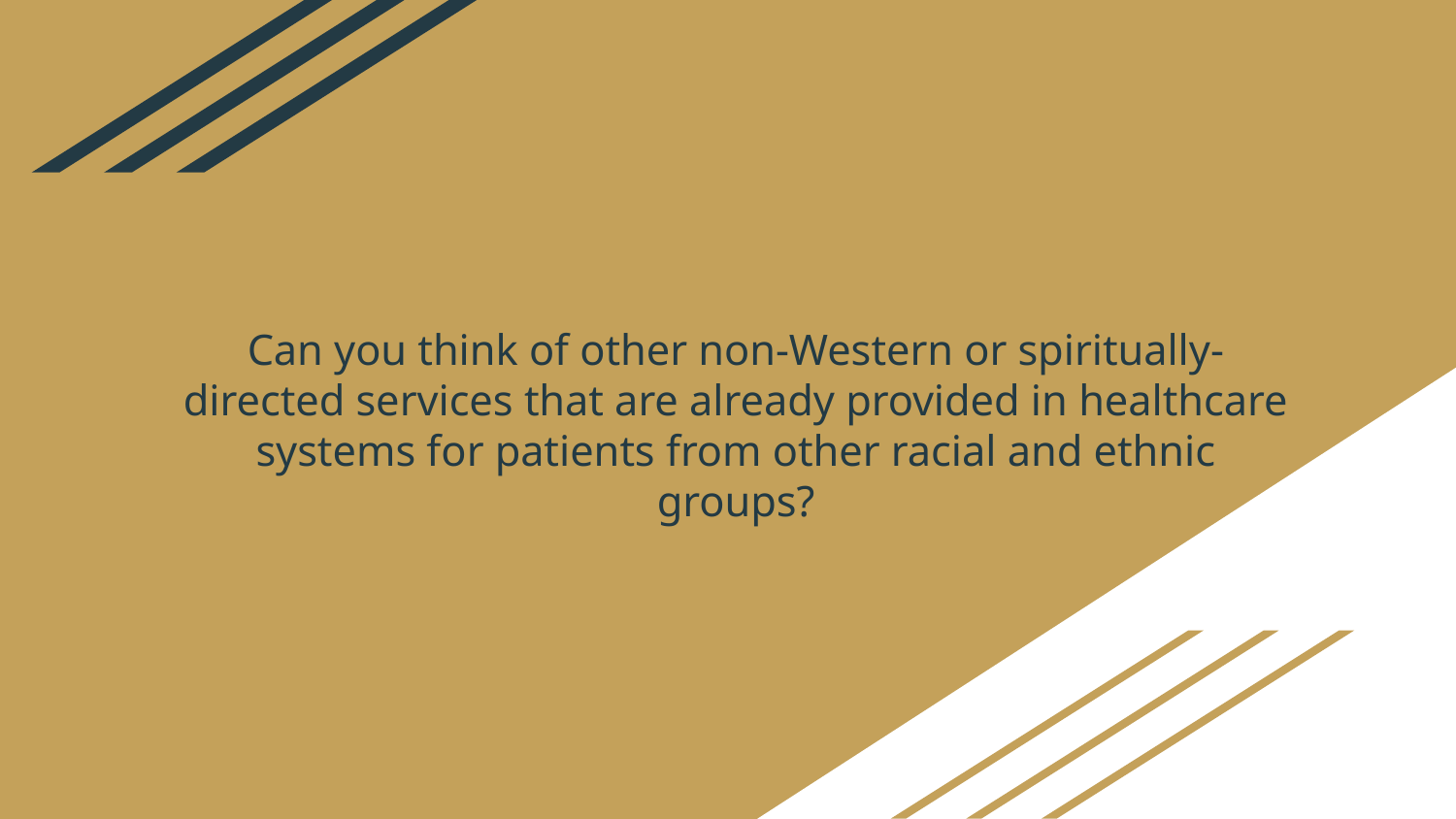

# Can you think of other non-Western or spiritually-directed services that are already provided in healthcare systems for patients from other racial and ethnic groups?

## Slide 16
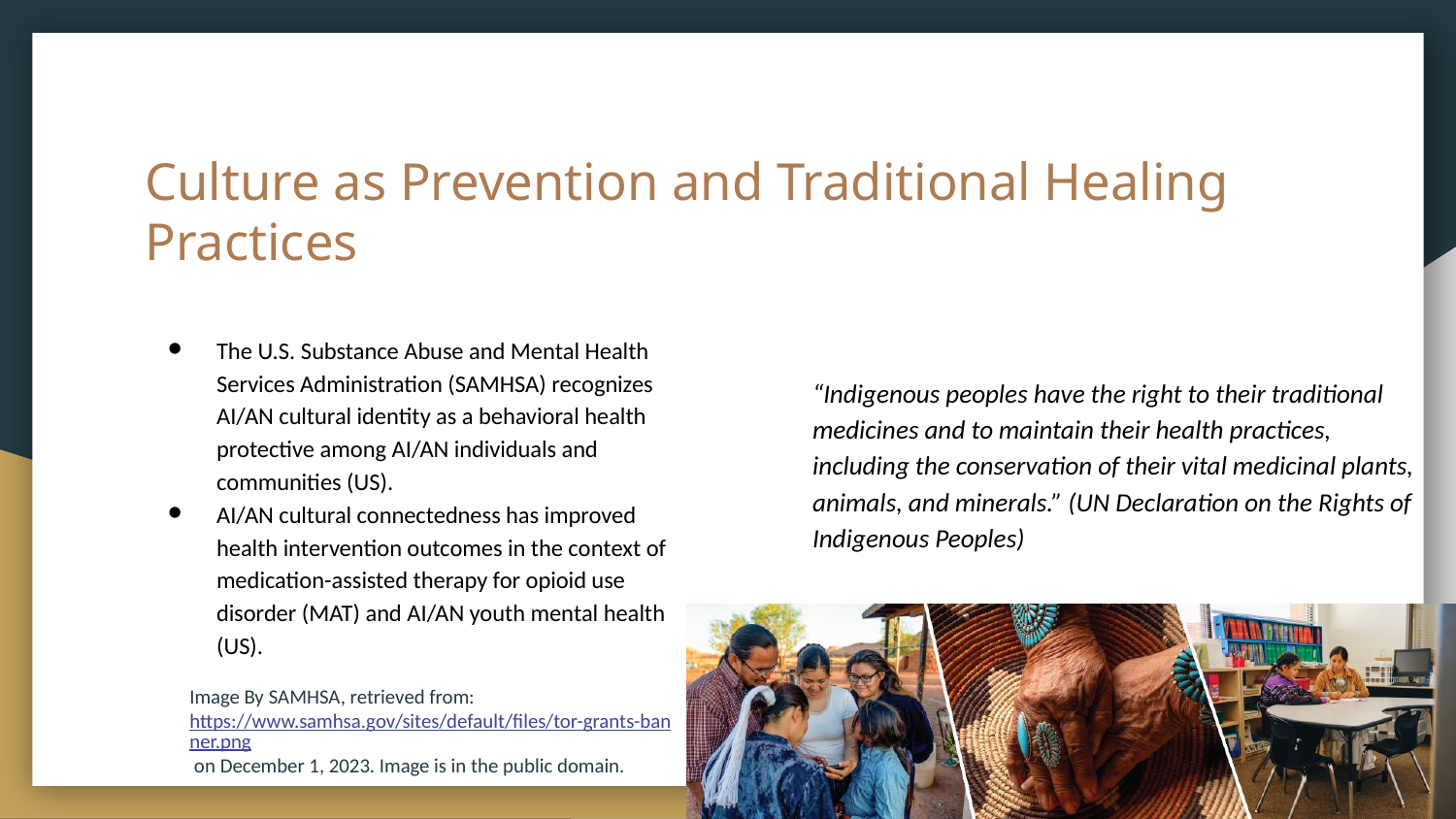

# Culture as Prevention and Traditional Healing Practices
The U.S. Substance Abuse and Mental Health Services Administration (SAMHSA) recognizes AI/AN cultural identity as a behavioral health protective among AI/AN individuals and communities (US).
AI/AN cultural connectedness has improved health intervention outcomes in the context of medication-assisted therapy for opioid use disorder (MAT) and AI/AN youth mental health (US).
“Indigenous peoples have the right to their traditional medicines and to maintain their health practices, including the conservation of their vital medicinal plants, animals, and minerals.” (UN Declaration on the Rights of Indigenous Peoples)
Image By SAMHSA, retrieved from: https://www.samhsa.gov/sites/default/files/tor-grants-banner.png on December 1, 2023. Image is in the public domain.

## Slide 17
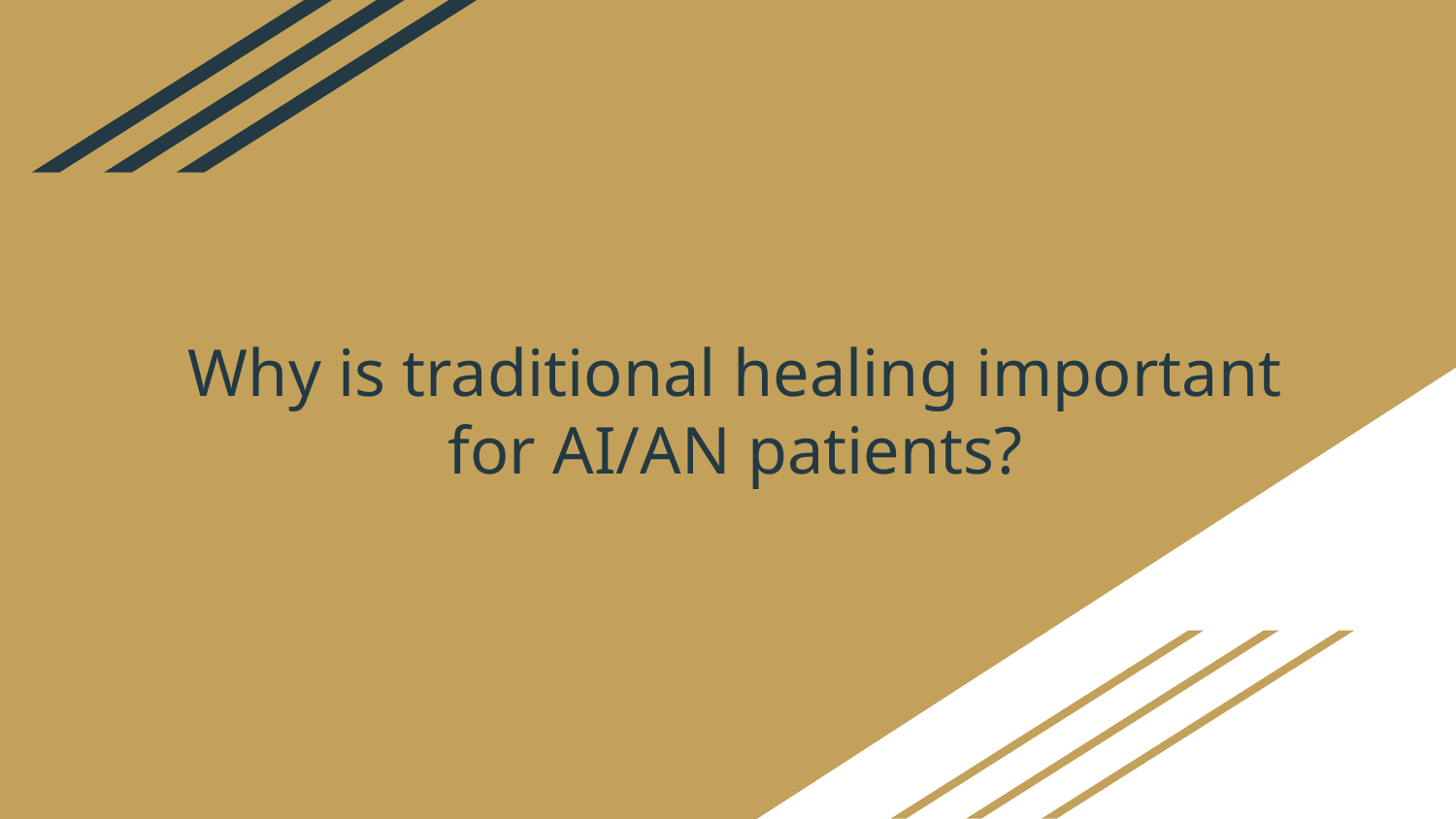

# Why is traditional healing important for AI/AN patients?

## Slide 18
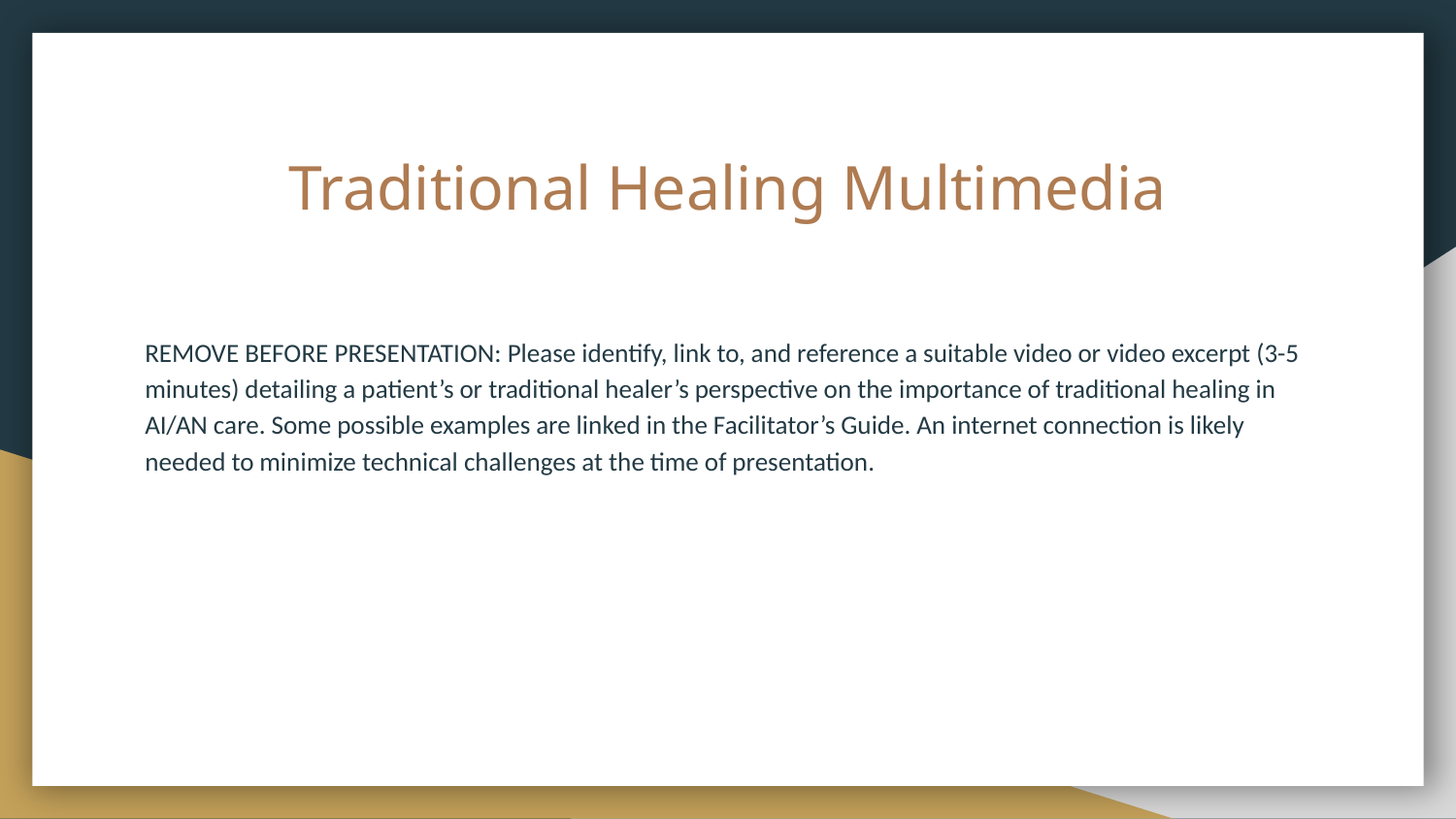

# Traditional Healing Multimedia
REMOVE BEFORE PRESENTATION: Please identify, link to, and reference a suitable video or video excerpt (3-5 minutes) detailing a patient’s or traditional healer’s perspective on the importance of traditional healing in AI/AN care. Some possible examples are linked in the Facilitator’s Guide. An internet connection is likely needed to minimize technical challenges at the time of presentation.

## Slide 19
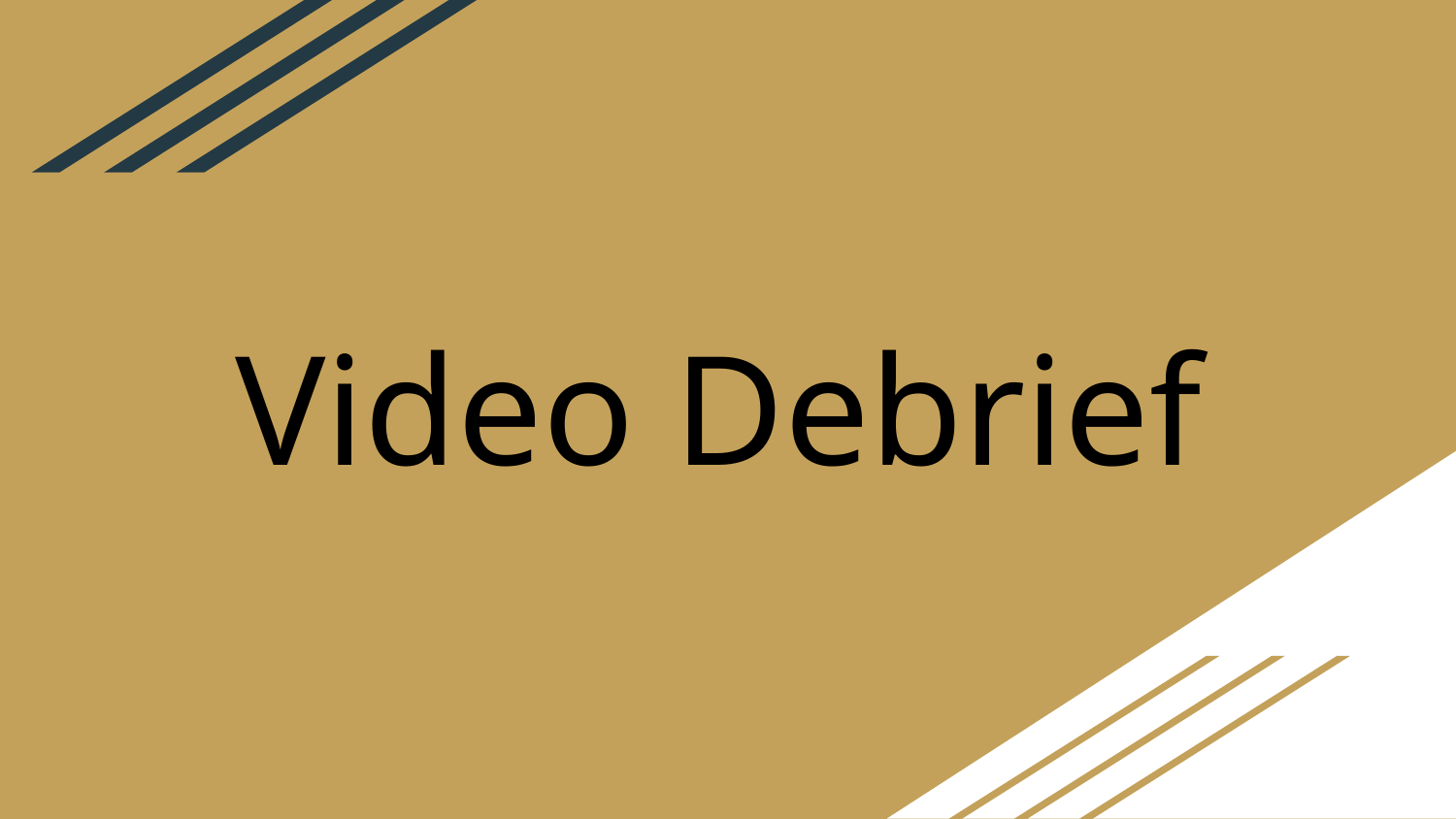

# Video Debrief

## Slide 20
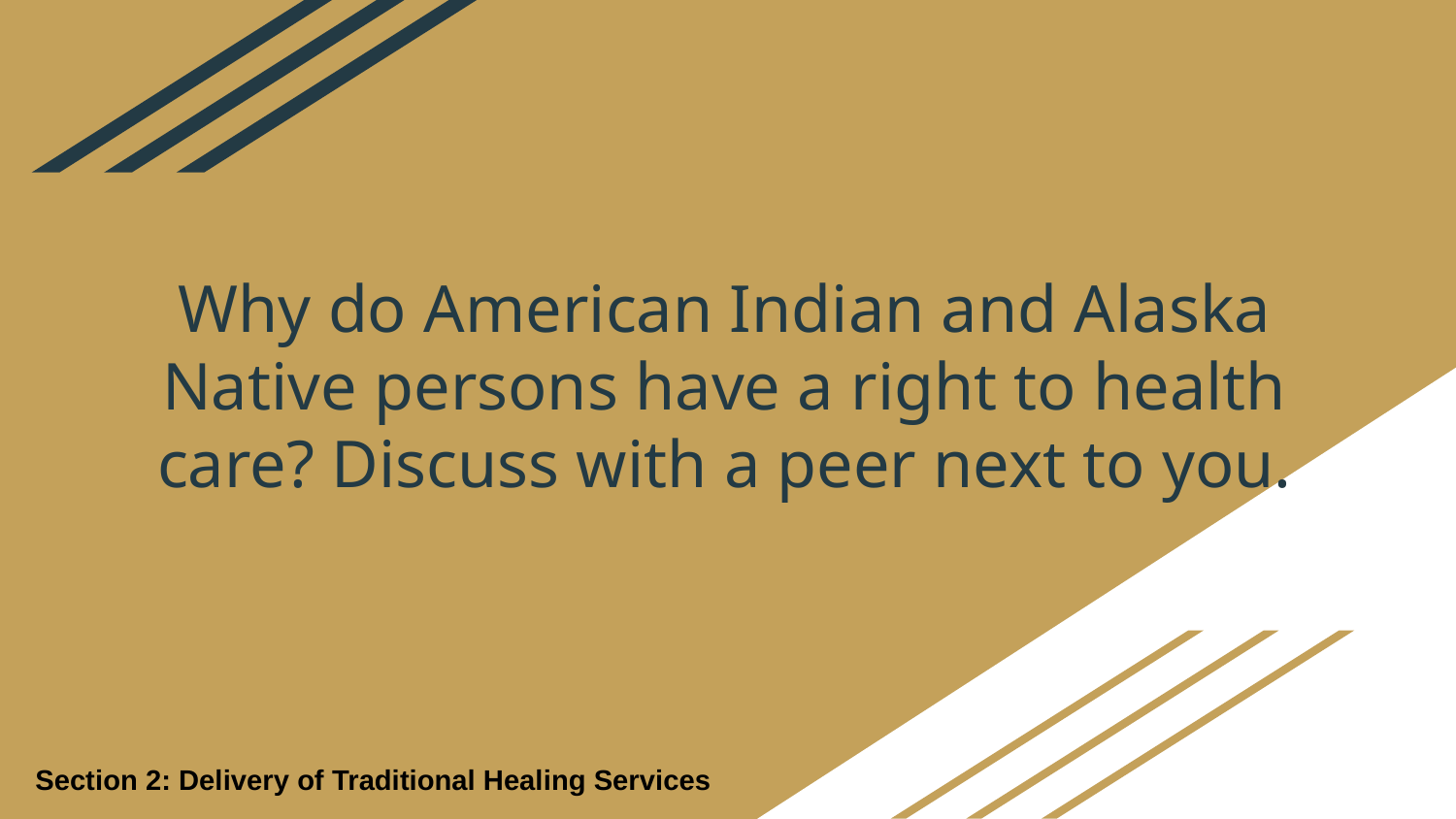

# Why do American Indian and Alaska Native persons have a right to health care? Discuss with a peer next to you.
Section 2: Delivery of Traditional Healing Services

## Slide 21
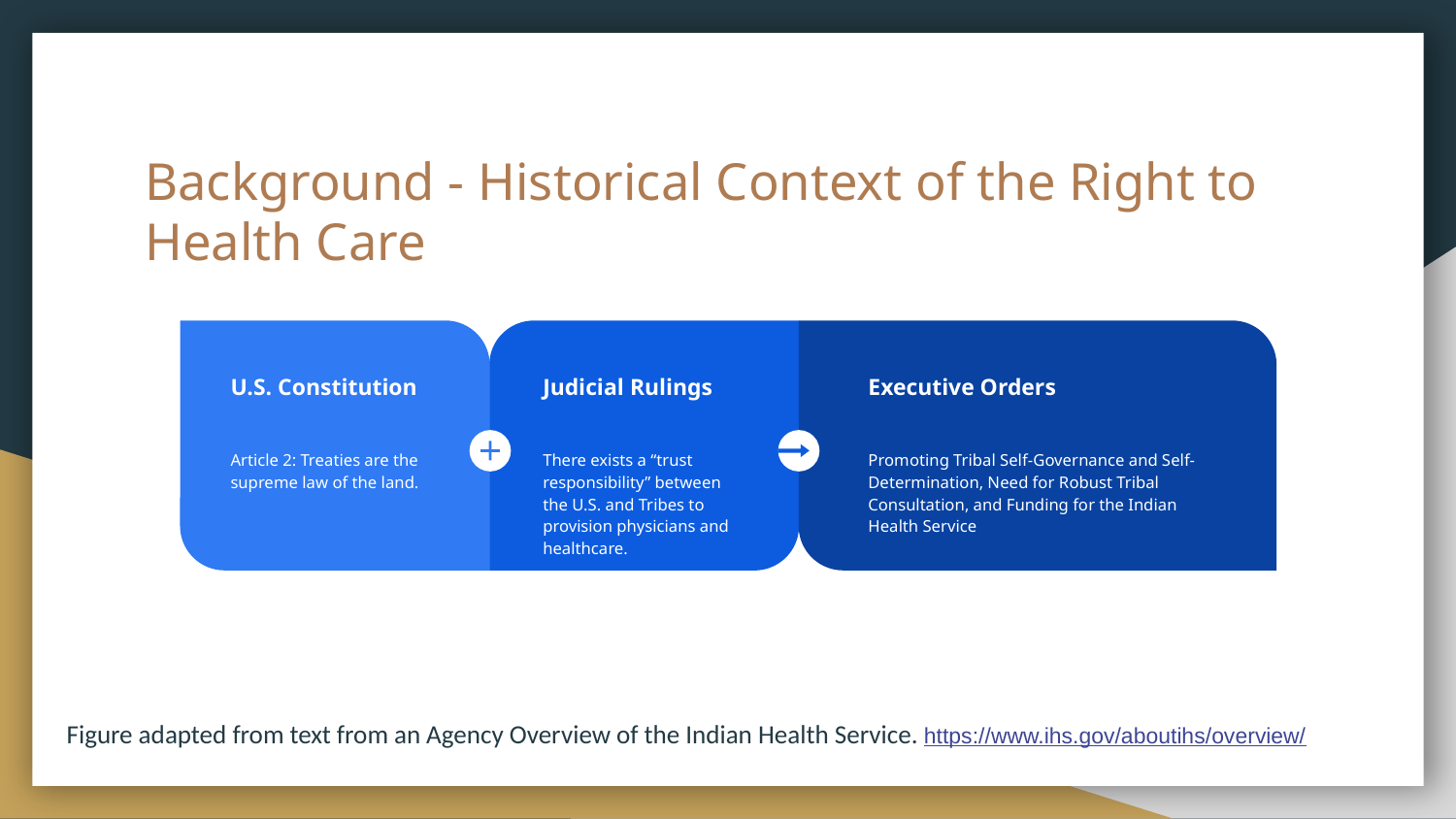

# Background - Historical Context of the Right to Health Care
U.S. Constitution
Article 2: Treaties are the supreme law of the land.
Judicial Rulings
There exists a “trust responsibility” between the U.S. and Tribes to provision physicians and healthcare.
Executive Orders
Promoting Tribal Self-Governance and Self-Determination, Need for Robust Tribal Consultation, and Funding for the Indian Health Service
Figure adapted from text from an Agency Overview of the Indian Health Service. https://www.ihs.gov/aboutihs/overview/

## Slide 22
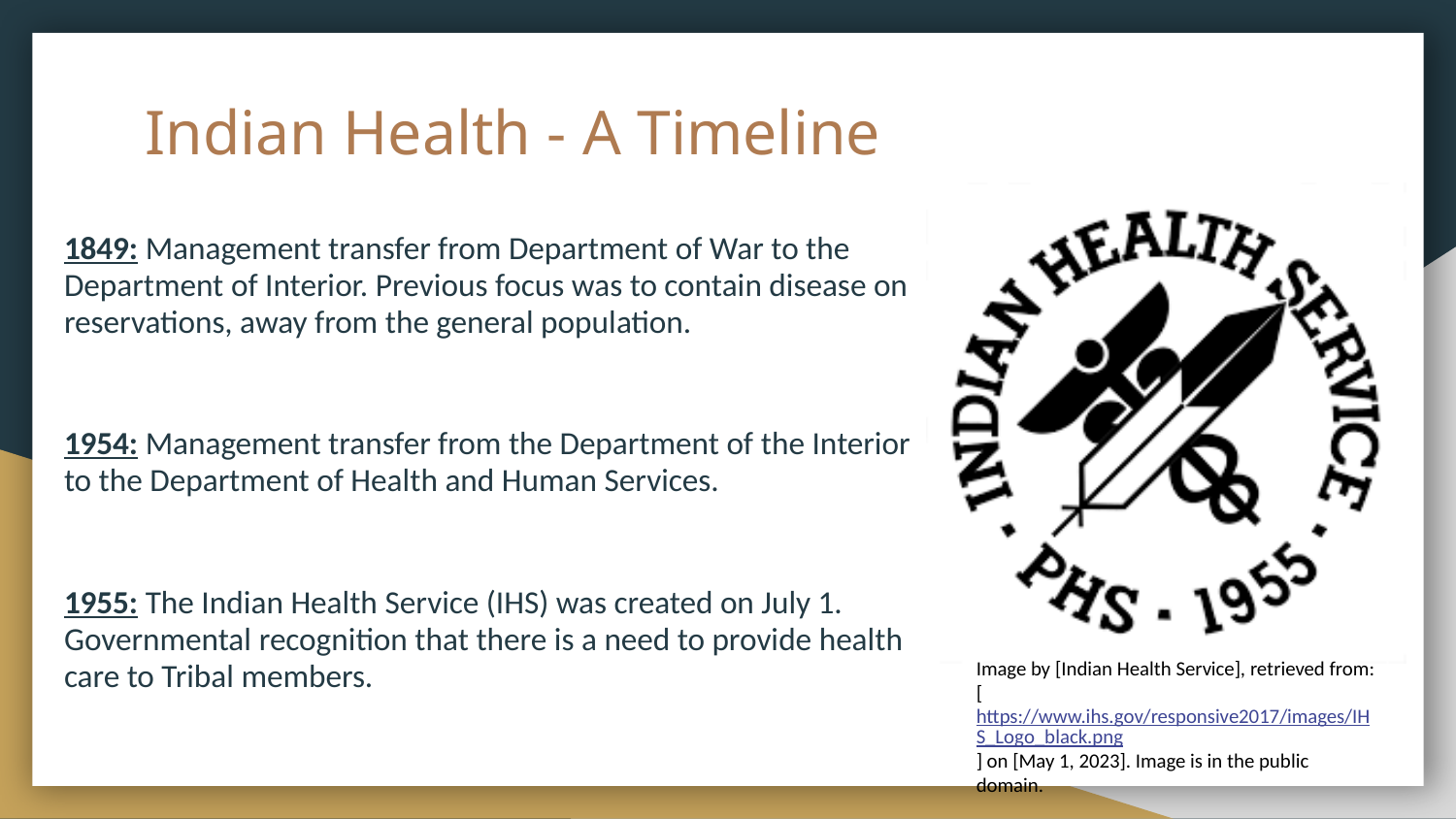

# Indian Health - A Timeline
1849: Management transfer from Department of War to the Department of Interior. Previous focus was to contain disease on reservations, away from the general population.
1954: Management transfer from the Department of the Interior to the Department of Health and Human Services.
1955: The Indian Health Service (IHS) was created on July 1. Governmental recognition that there is a need to provide health care to Tribal members.
Image by [Indian Health Service], retrieved from: [https://www.ihs.gov/responsive2017/images/IHS_Logo_black.png] on [May 1, 2023]. Image is in the public domain.

## Slide 23
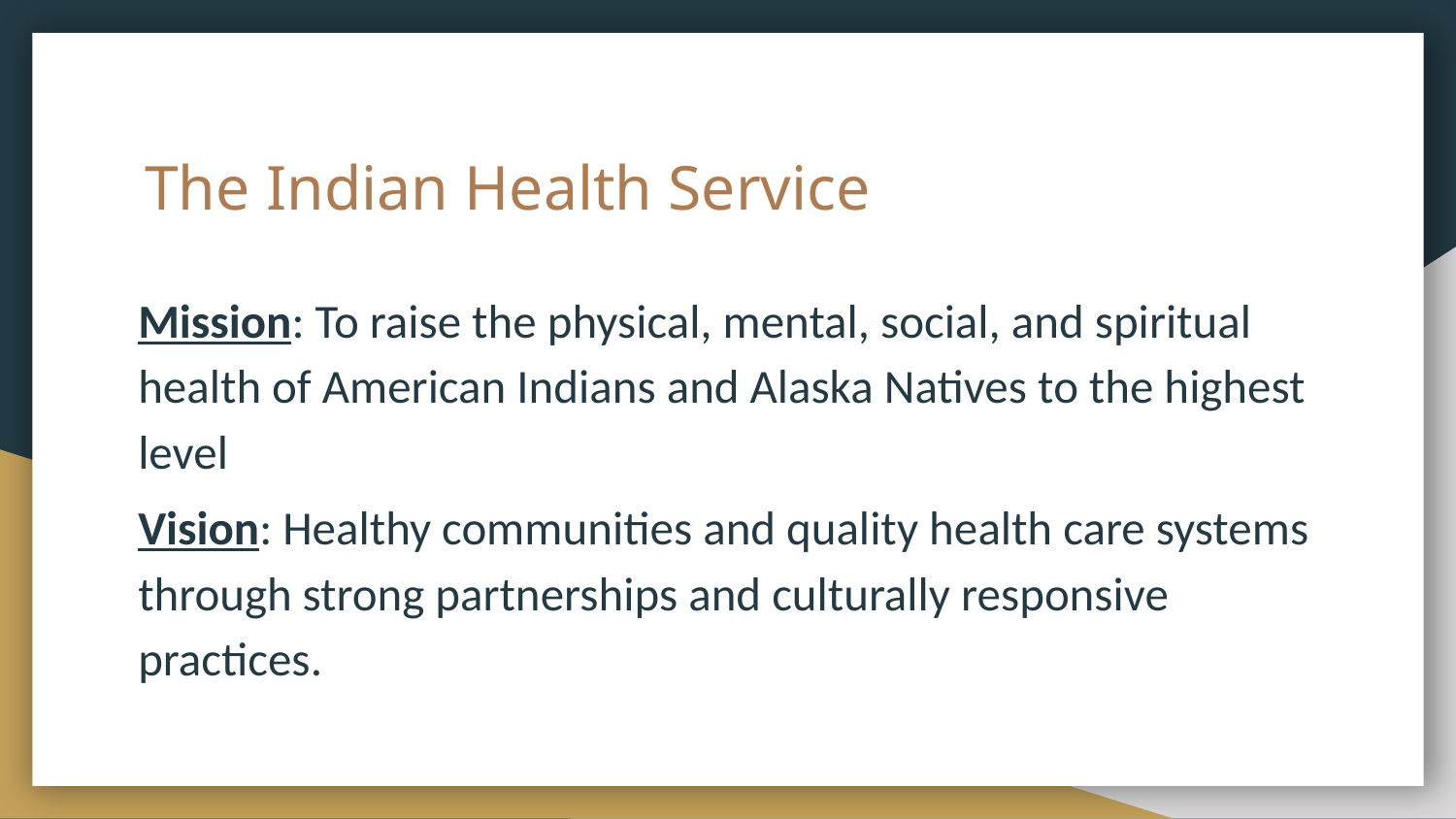

# The Indian Health Service
Mission: To raise the physical, mental, social, and spiritual health of American Indians and Alaska Natives to the highest level
Vision: Healthy communities and quality health care systems through strong partnerships and culturally responsive practices.

## Slide 24
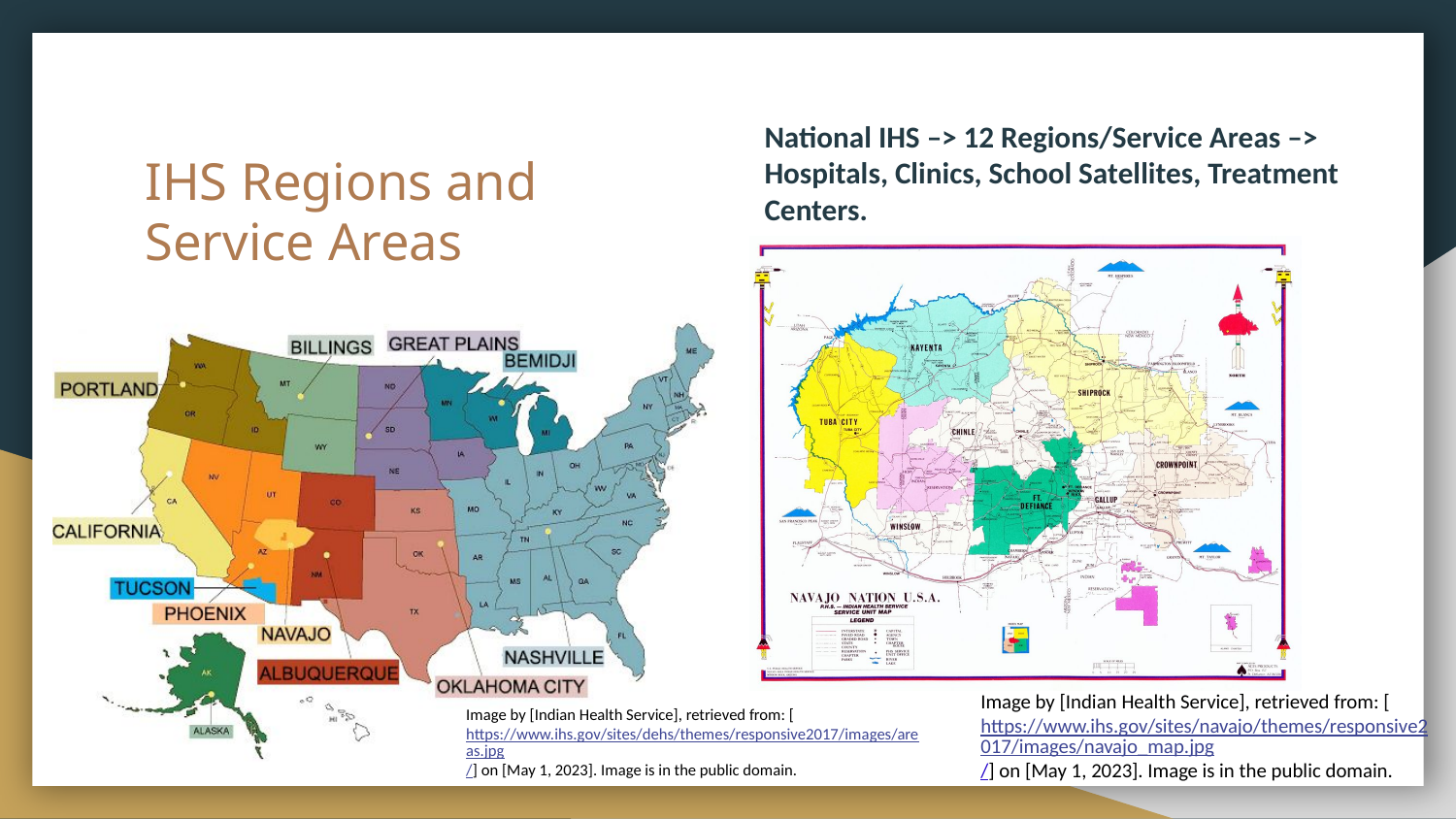

National IHS –> 12 Regions/Service Areas –> Hospitals, Clinics, School Satellites, Treatment Centers.
# IHS Regions and Service Areas
Image by [Indian Health Service], retrieved from: [https://www.ihs.gov/sites/navajo/themes/responsive2017/images/navajo_map.jpg/] on [May 1, 2023]. Image is in the public domain.
Image by [Indian Health Service], retrieved from: [https://www.ihs.gov/sites/dehs/themes/responsive2017/images/areas.jpg/] on [May 1, 2023]. Image is in the public domain.

## Slide 25
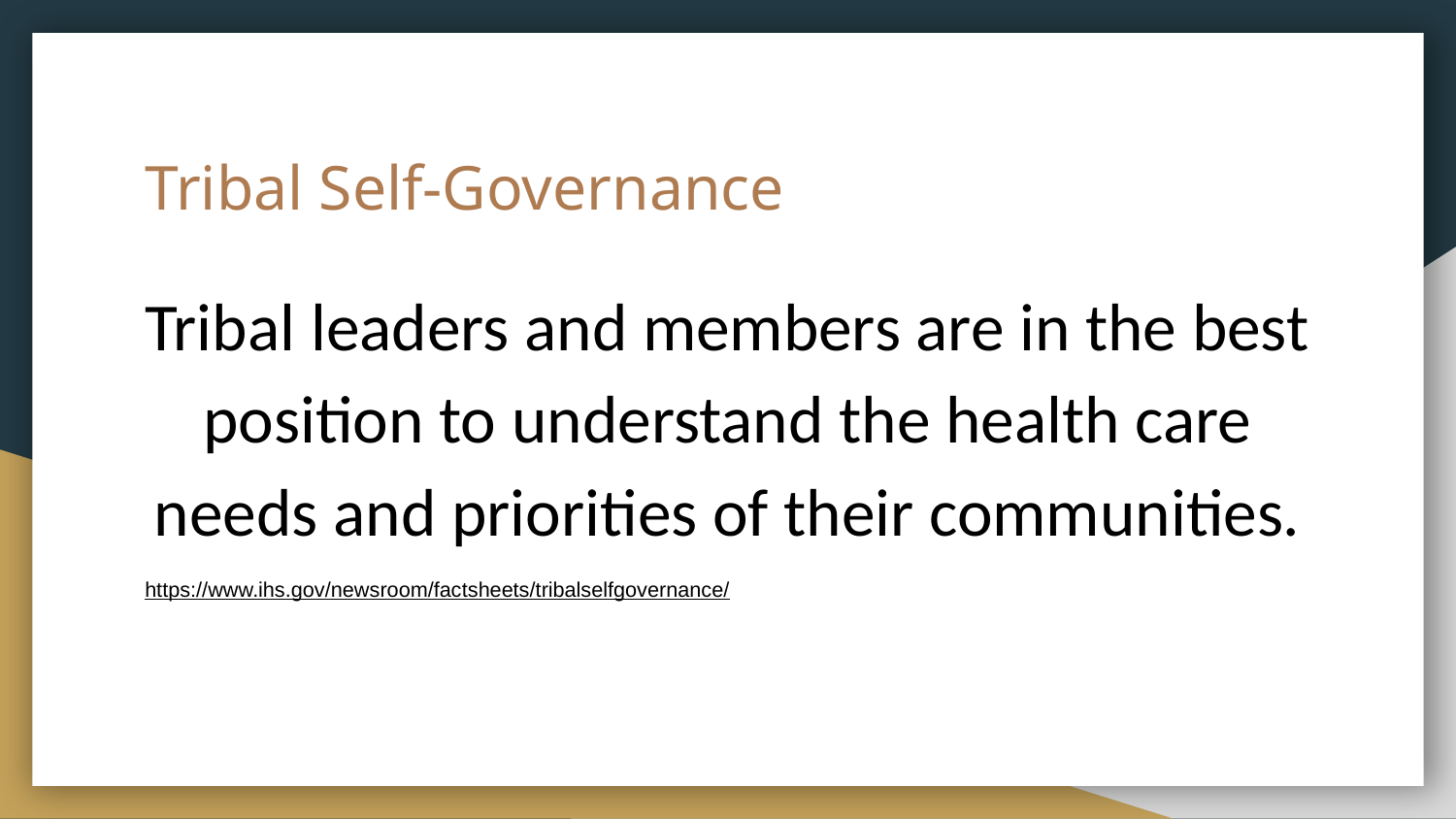

# Tribal Self-Governance
Tribal leaders and members are in the best position to understand the health care needs and priorities of their communities.
https://www.ihs.gov/newsroom/factsheets/tribalselfgovernance/

## Slide 26
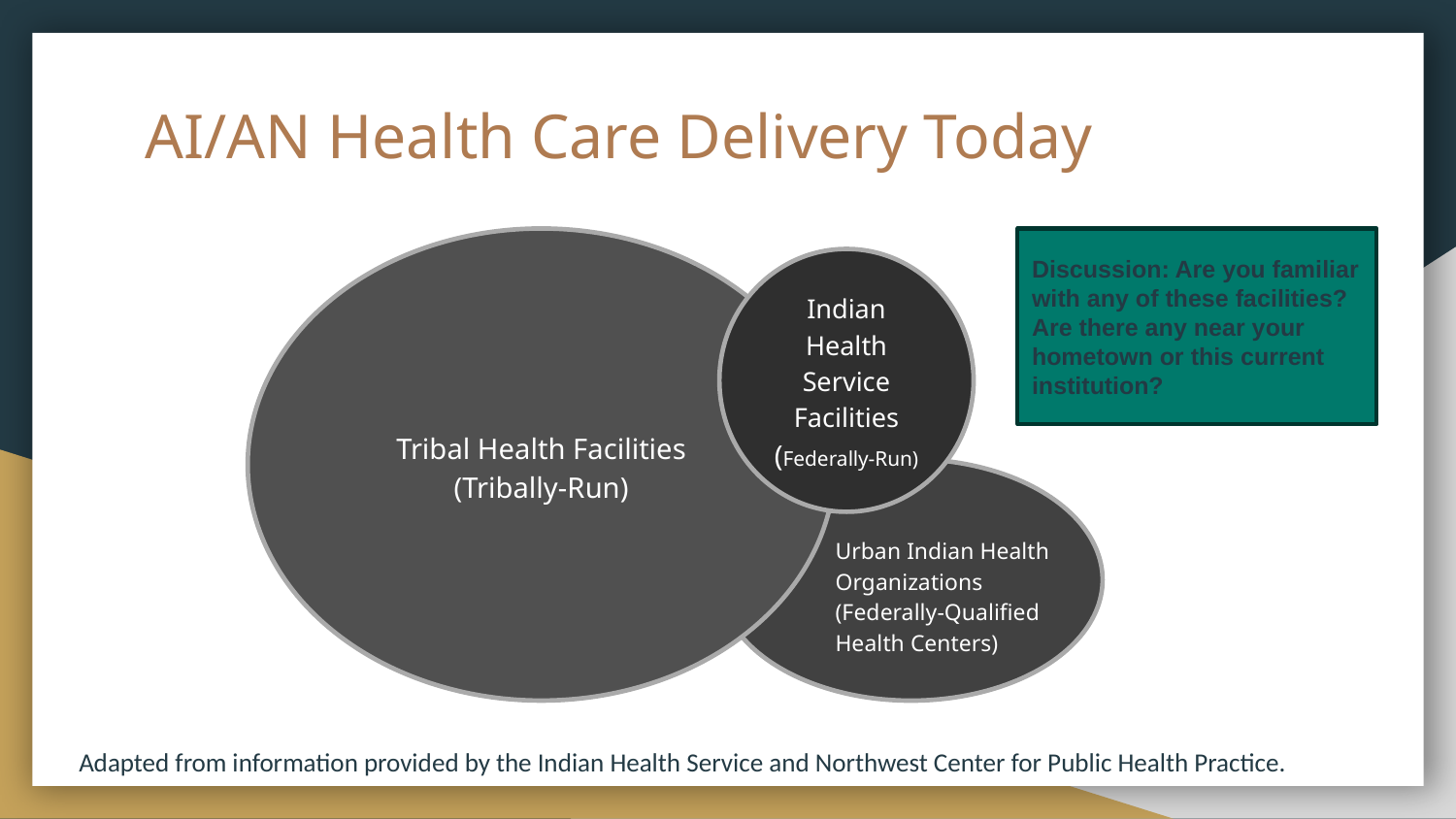

# AI/AN Health Care Delivery Today
Tribal Health Facilities (Tribally-Run)
Discussion: Are you familiar with any of these facilities? Are there any near your hometown or this current institution?
Indian Health Service Facilities (Federally-Run)
Urban Indian Health Organizations (Federally-Qualified Health Centers)
Adapted from information provided by the Indian Health Service and Northwest Center for Public Health Practice.

## Slide 27
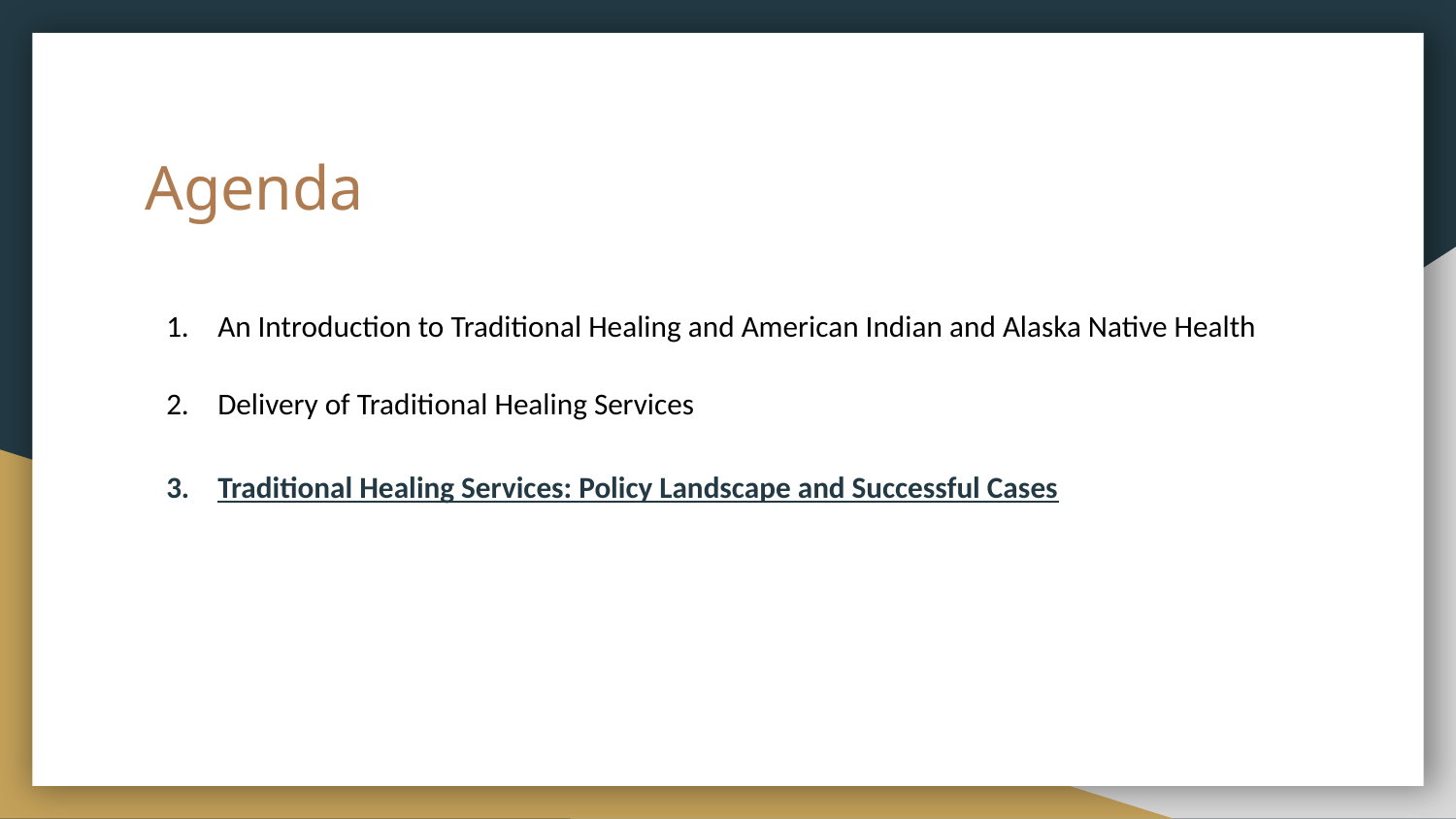

# Agenda
An Introduction to Traditional Healing and American Indian and Alaska Native Health
Delivery of Traditional Healing Services
Traditional Healing Services: Policy Landscape and Successful Cases

## Slide 28
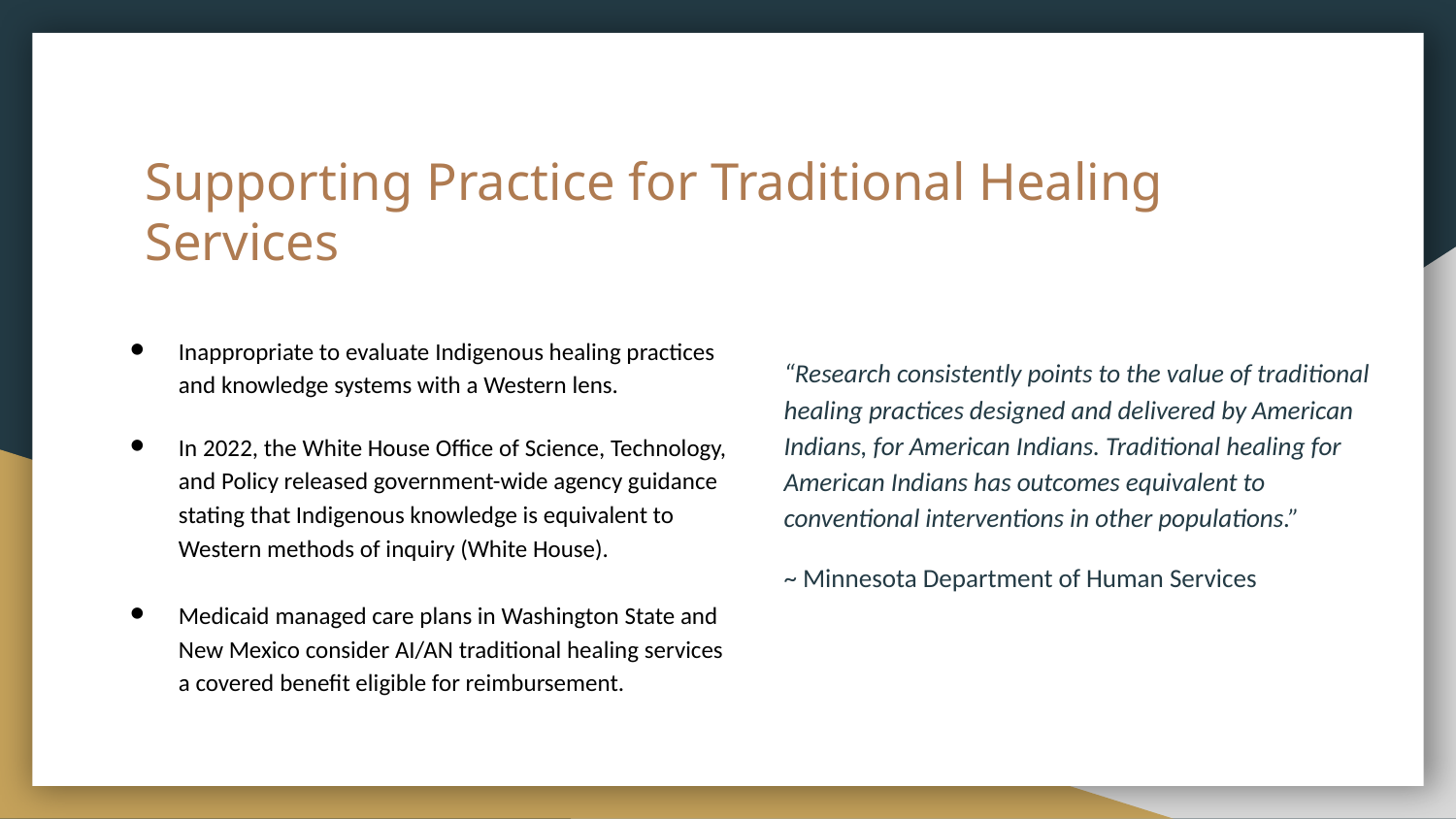

# Supporting Practice for Traditional Healing Services
Inappropriate to evaluate Indigenous healing practices and knowledge systems with a Western lens.
In 2022, the White House Office of Science, Technology, and Policy released government-wide agency guidance stating that Indigenous knowledge is equivalent to Western methods of inquiry (White House).
Medicaid managed care plans in Washington State and New Mexico consider AI/AN traditional healing services a covered benefit eligible for reimbursement.
“Research consistently points to the value of traditional healing practices designed and delivered by American Indians, for American Indians. Traditional healing for American Indians has outcomes equivalent to conventional interventions in other populations.”
~ Minnesota Department of Human Services

## Slide 29
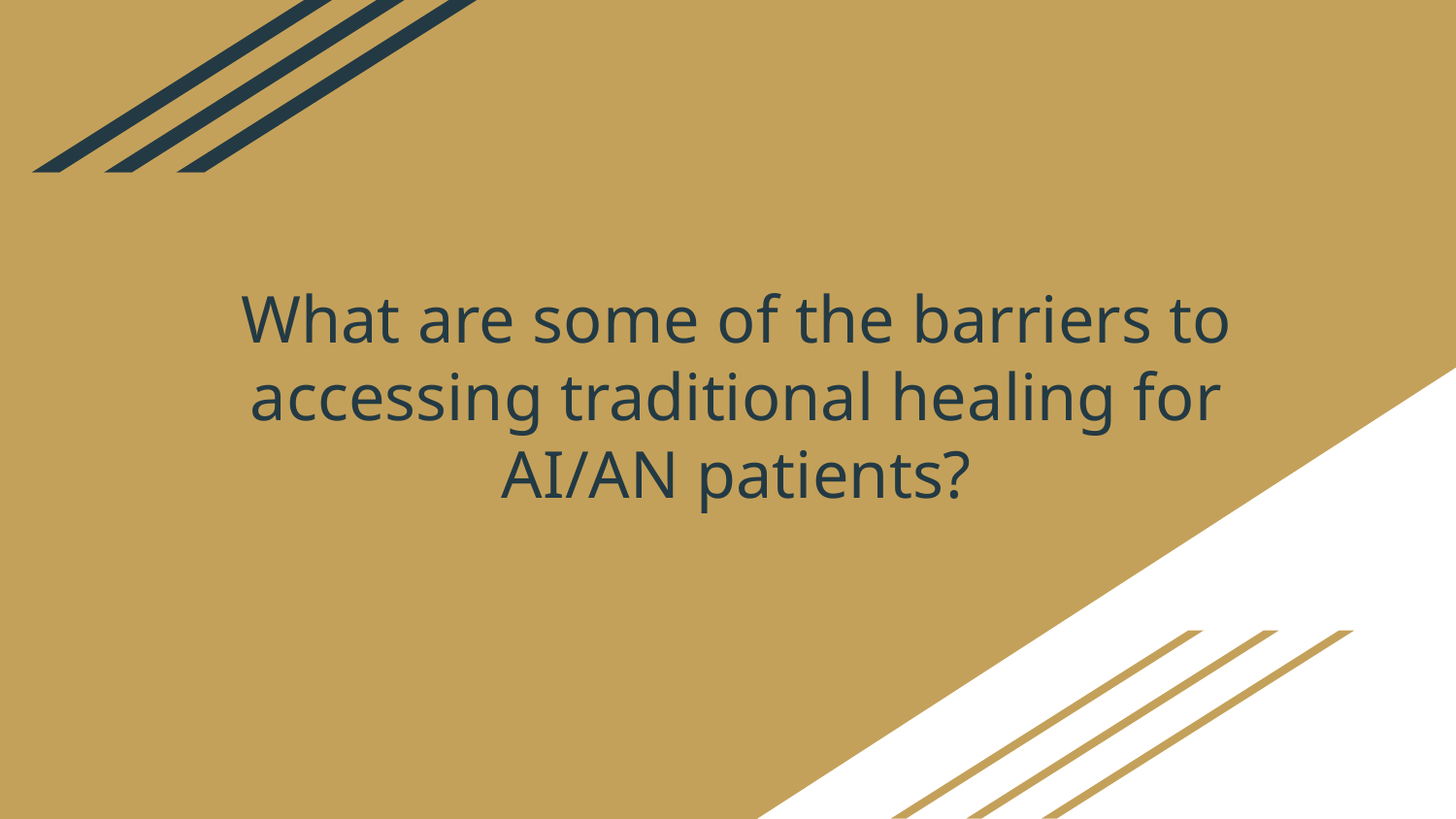

# What are some of the barriers to accessing traditional healing for AI/AN patients?

## Slide 30
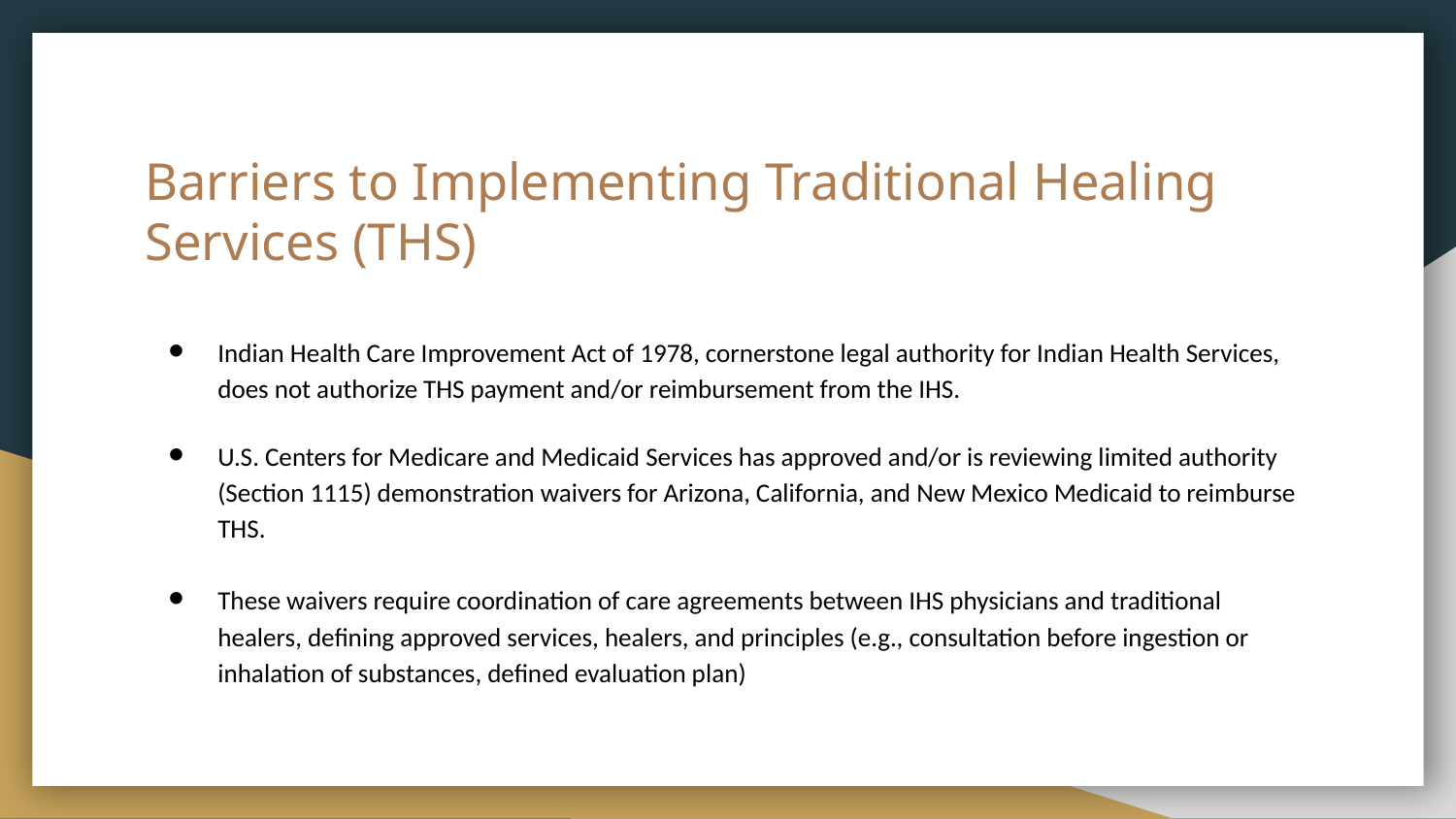

# Barriers to Implementing Traditional Healing Services (THS)
Indian Health Care Improvement Act of 1978, cornerstone legal authority for Indian Health Services, does not authorize THS payment and/or reimbursement from the IHS.
U.S. Centers for Medicare and Medicaid Services has approved and/or is reviewing limited authority (Section 1115) demonstration waivers for Arizona, California, and New Mexico Medicaid to reimburse THS.
These waivers require coordination of care agreements between IHS physicians and traditional healers, defining approved services, healers, and principles (e.g., consultation before ingestion or inhalation of substances, defined evaluation plan)

## Slide 31
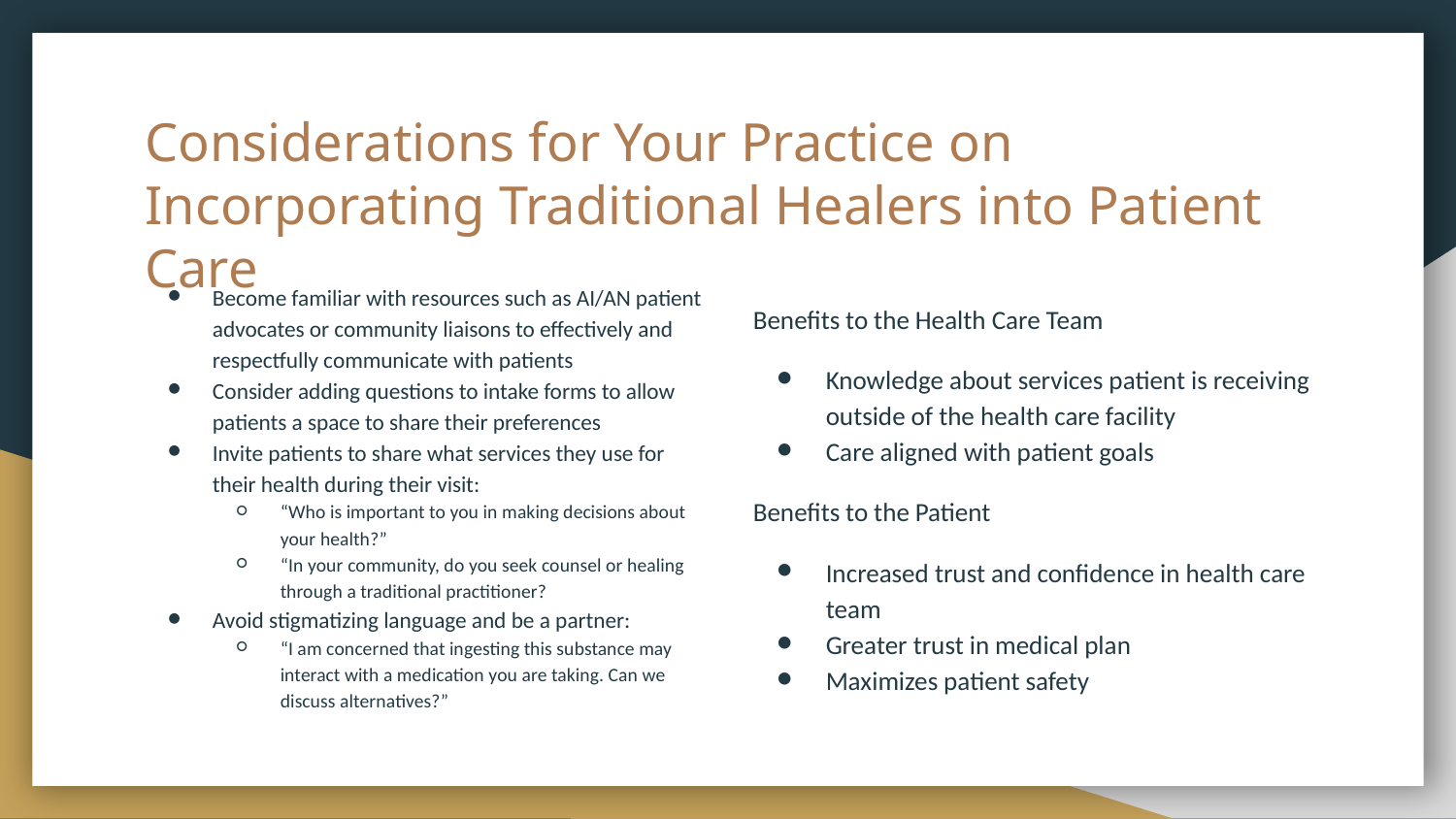

# Considerations for Your Practice on Incorporating Traditional Healers into Patient Care
Become familiar with resources such as AI/AN patient advocates or community liaisons to effectively and respectfully communicate with patients
Consider adding questions to intake forms to allow patients a space to share their preferences
Invite patients to share what services they use for their health during their visit:
“Who is important to you in making decisions about your health?”
“In your community, do you seek counsel or healing through a traditional practitioner?
Avoid stigmatizing language and be a partner:
“I am concerned that ingesting this substance may interact with a medication you are taking. Can we discuss alternatives?”
Benefits to the Health Care Team
Knowledge about services patient is receiving outside of the health care facility
Care aligned with patient goals
Benefits to the Patient
Increased trust and confidence in health care team
Greater trust in medical plan
Maximizes patient safety

## Slide 32
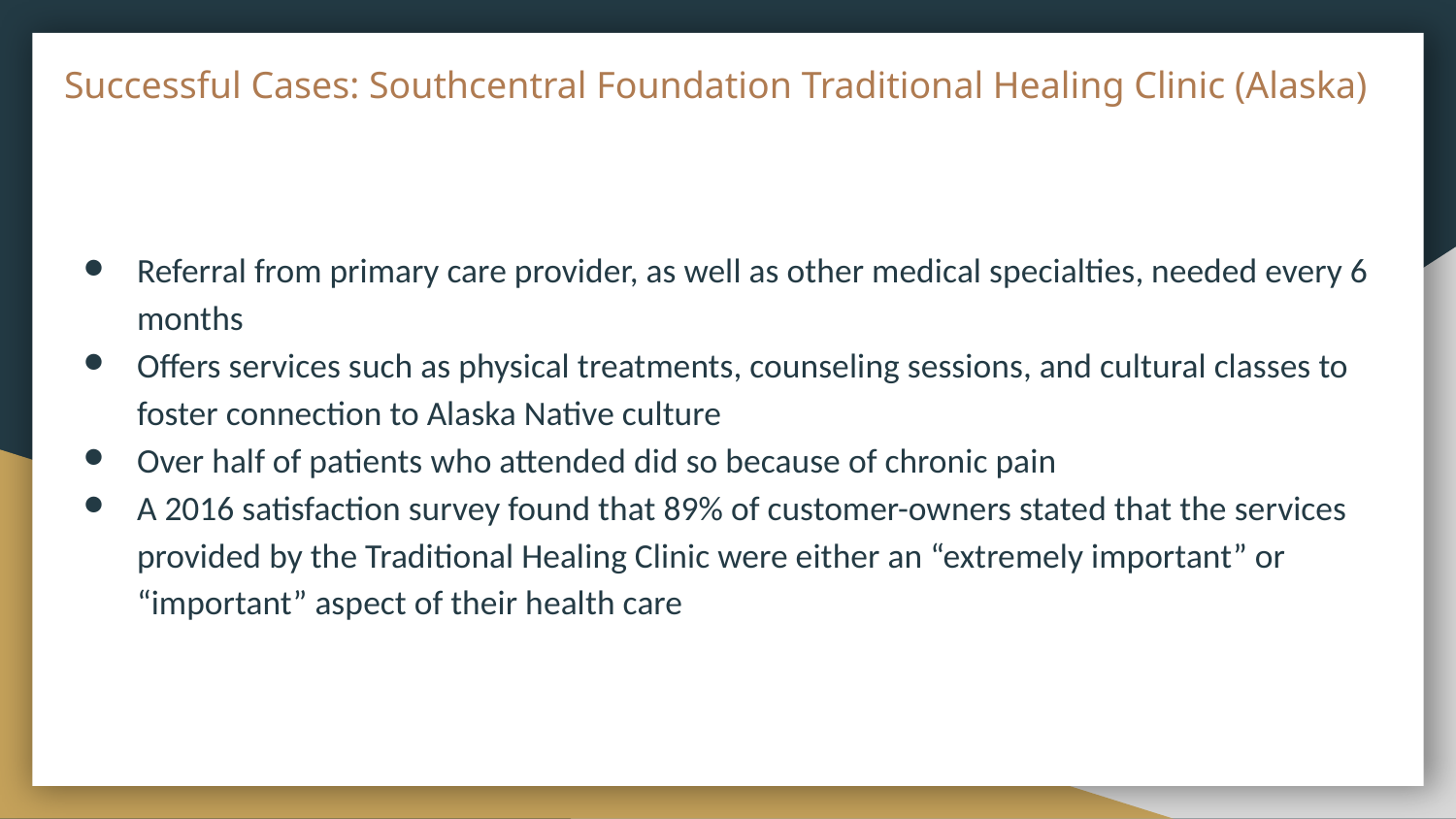

# Successful Cases: Southcentral Foundation Traditional Healing Clinic (Alaska)
Referral from primary care provider, as well as other medical specialties, needed every 6 months
Offers services such as physical treatments, counseling sessions, and cultural classes to foster connection to Alaska Native culture
Over half of patients who attended did so because of chronic pain
A 2016 satisfaction survey found that 89% of customer-owners stated that the services provided by the Traditional Healing Clinic were either an “extremely important” or “important” aspect of their health care

## Slide 33
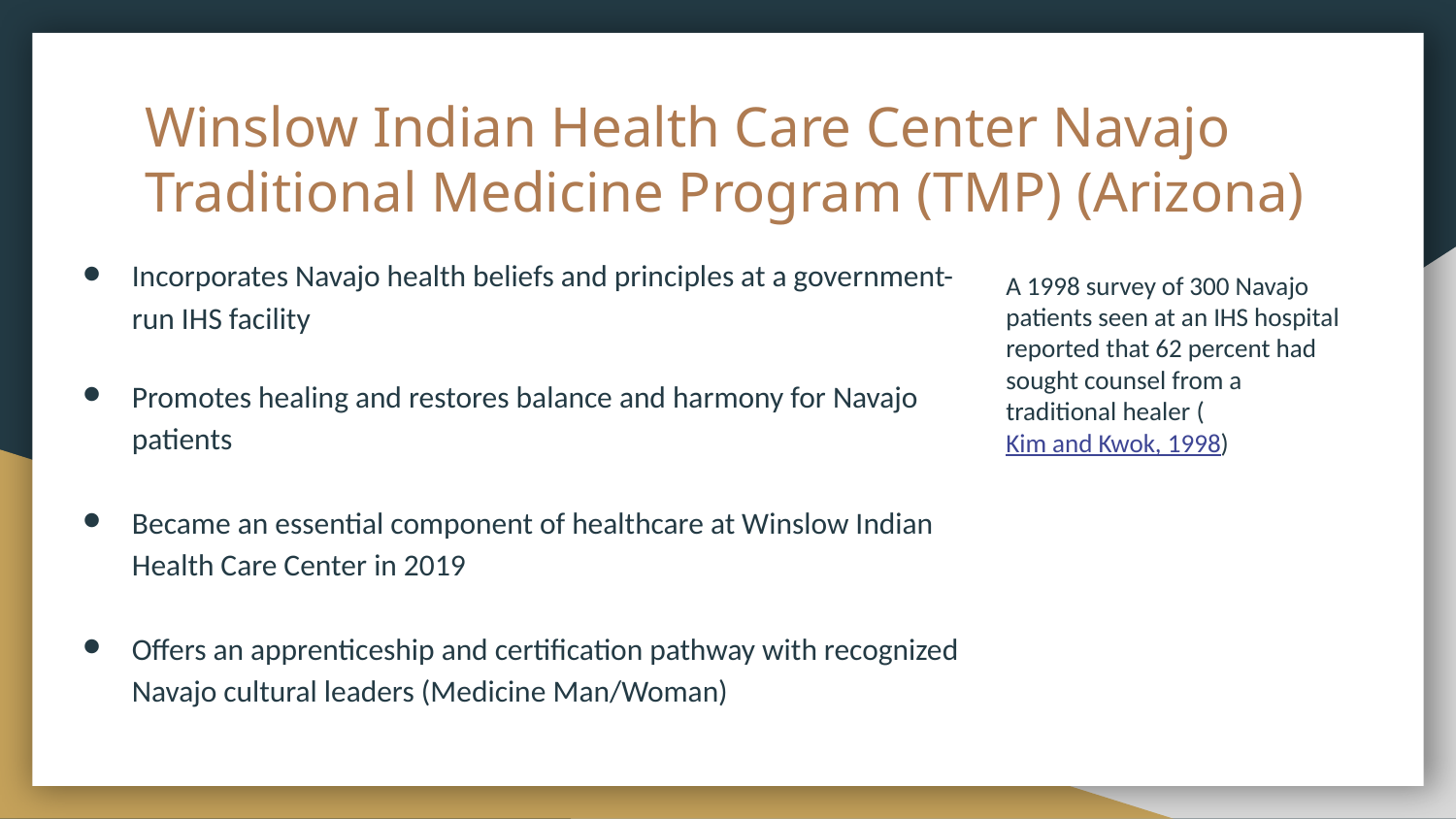

# Winslow Indian Health Care Center Navajo Traditional Medicine Program (TMP) (Arizona)
Incorporates Navajo health beliefs and principles at a government-run IHS facility
Promotes healing and restores balance and harmony for Navajo patients
Became an essential component of healthcare at Winslow Indian Health Care Center in 2019
Offers an apprenticeship and certification pathway with recognized Navajo cultural leaders (Medicine Man/Woman)
A 1998 survey of 300 Navajo patients seen at an IHS hospital reported that 62 percent had sought counsel from a traditional healer (Kim and Kwok, 1998)

## Slide 34
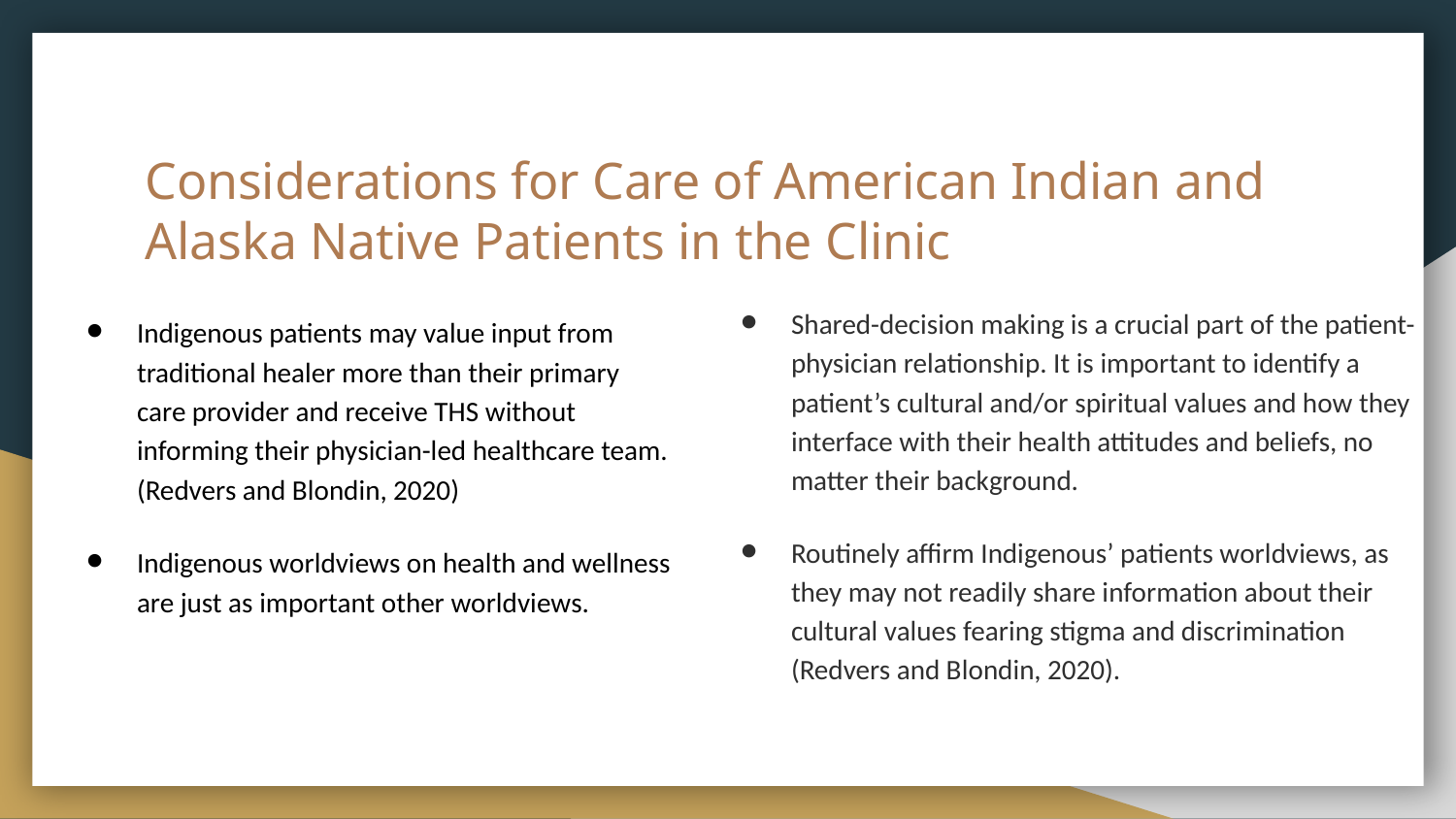

# Considerations for Care of American Indian and Alaska Native Patients in the Clinic
Shared-decision making is a crucial part of the patient-physician relationship. It is important to identify a patient’s cultural and/or spiritual values and how they interface with their health attitudes and beliefs, no matter their background.
Routinely affirm Indigenous’ patients worldviews, as they may not readily share information about their cultural values fearing stigma and discrimination (Redvers and Blondin, 2020).
Indigenous patients may value input from traditional healer more than their primary care provider and receive THS without informing their physician-led healthcare team. (Redvers and Blondin, 2020)
Indigenous worldviews on health and wellness are just as important other worldviews.

## Slide 35
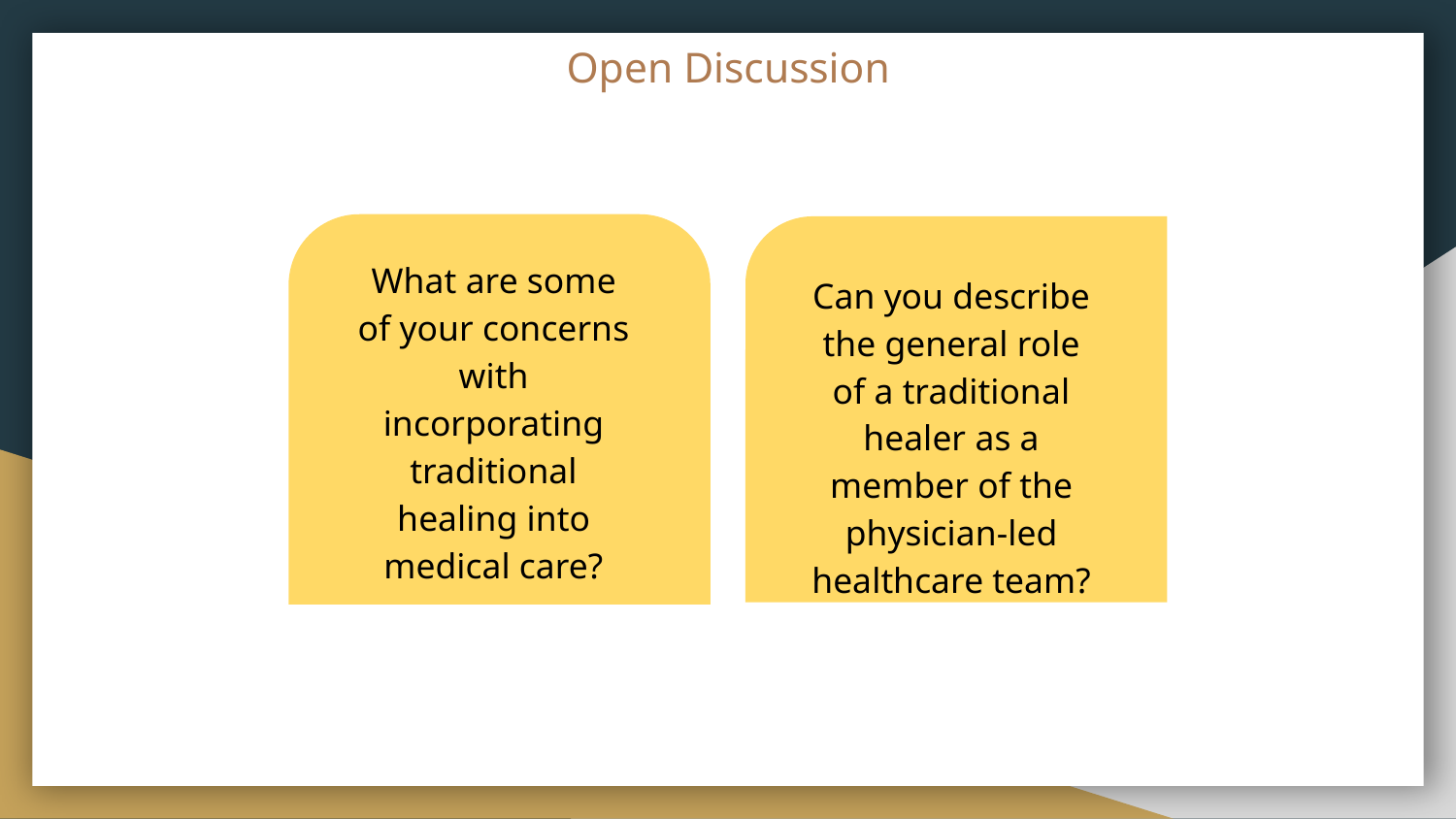

# Open Discussion
What are some of your concerns with incorporating traditional healing into medical care?
Can you describe the general role of a traditional healer as a member of the physician-led healthcare team?

## Slide 36
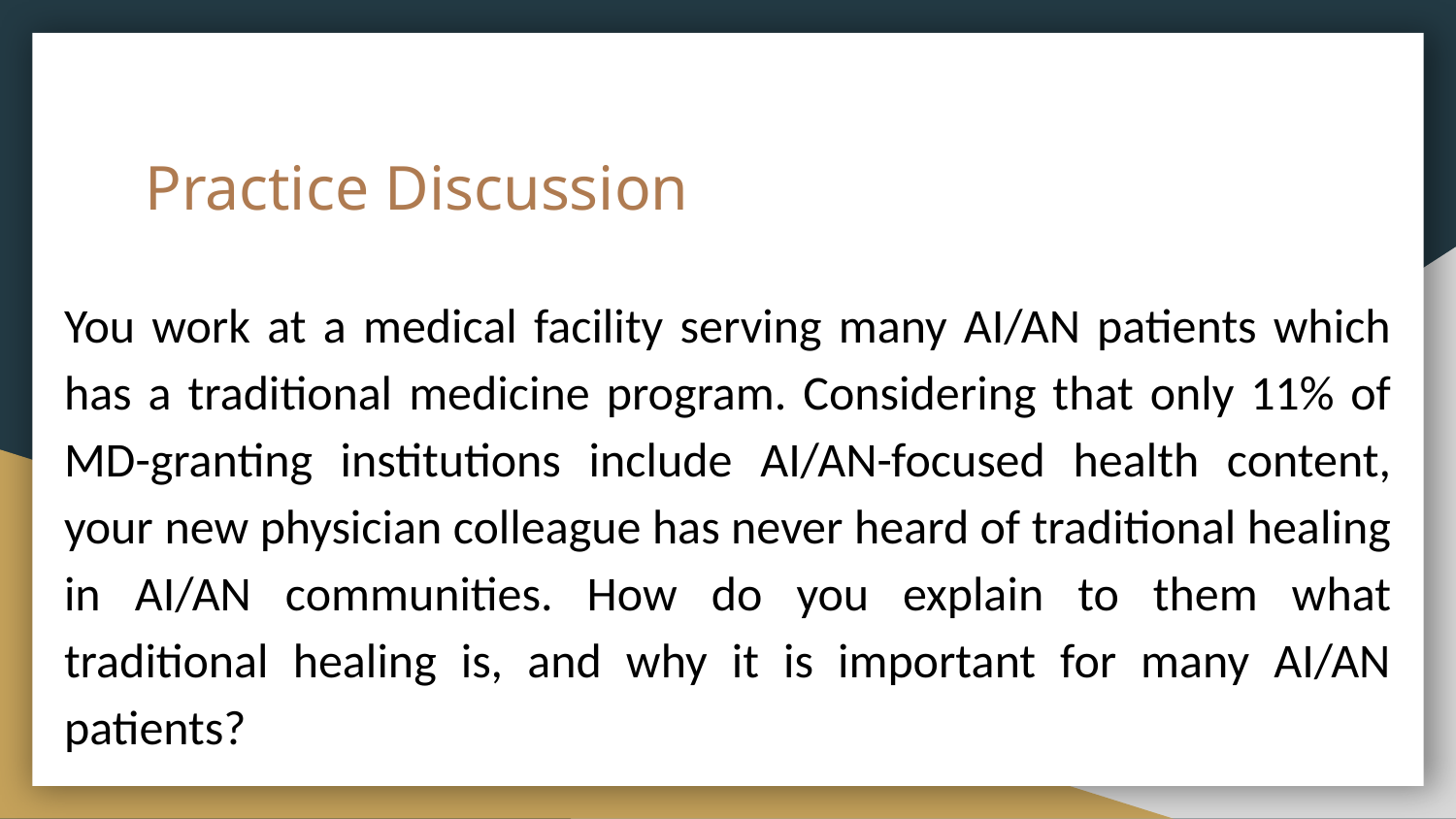

# Practice Discussion
You work at a medical facility serving many AI/AN patients which has a traditional medicine program. Considering that only 11% of MD-granting institutions include AI/AN-focused health content, your new physician colleague has never heard of traditional healing in AI/AN communities. How do you explain to them what traditional healing is, and why it is important for many AI/AN patients?

## Slide 37
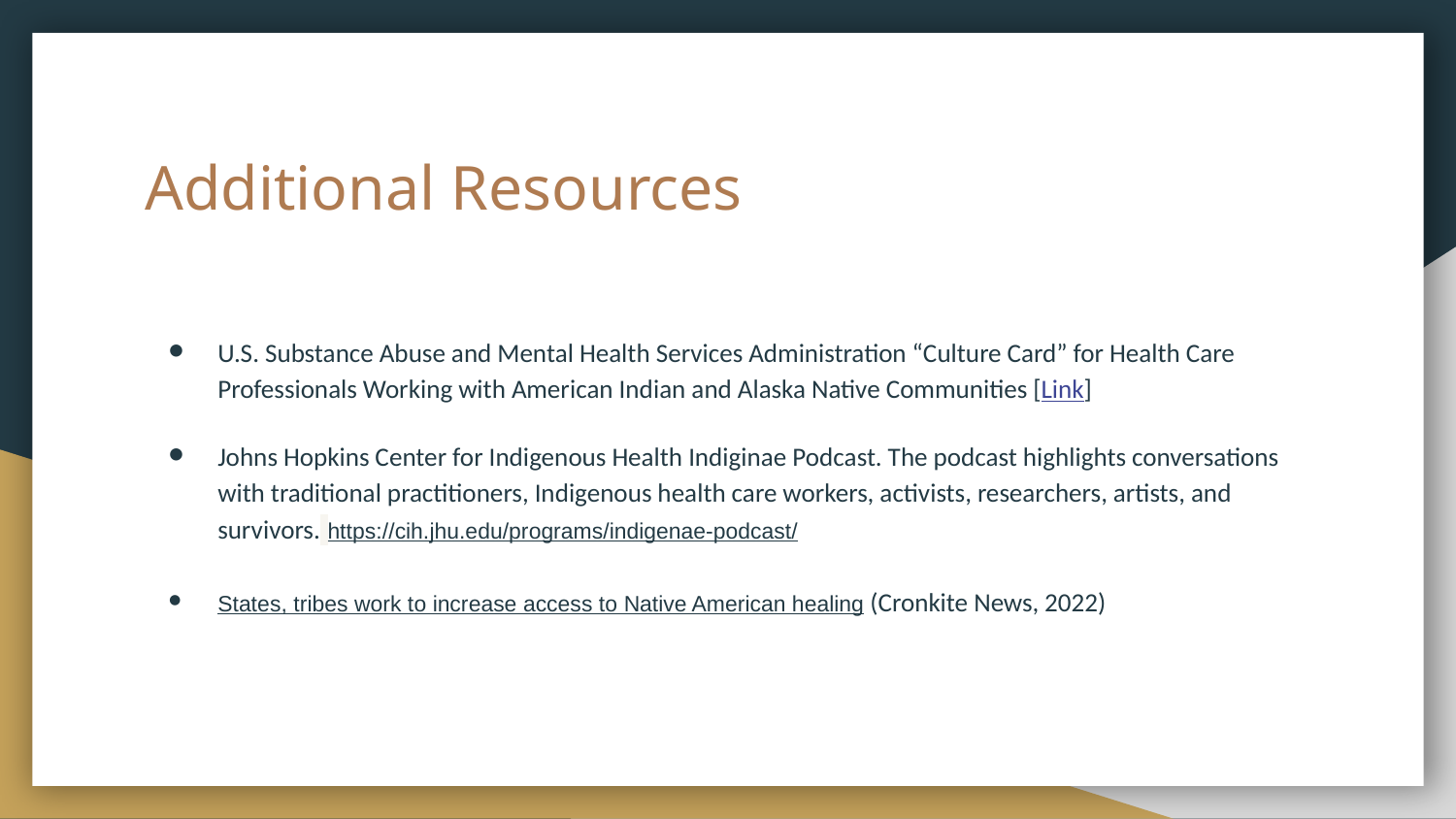

# Additional Resources
U.S. Substance Abuse and Mental Health Services Administration “Culture Card” for Health Care Professionals Working with American Indian and Alaska Native Communities [Link]
Johns Hopkins Center for Indigenous Health Indiginae Podcast. The podcast highlights conversations with traditional practitioners, Indigenous health care workers, activists, researchers, artists, and survivors. https://cih.jhu.edu/programs/indigenae-podcast/
States, tribes work to increase access to Native American healing (Cronkite News, 2022)

## Slide 38
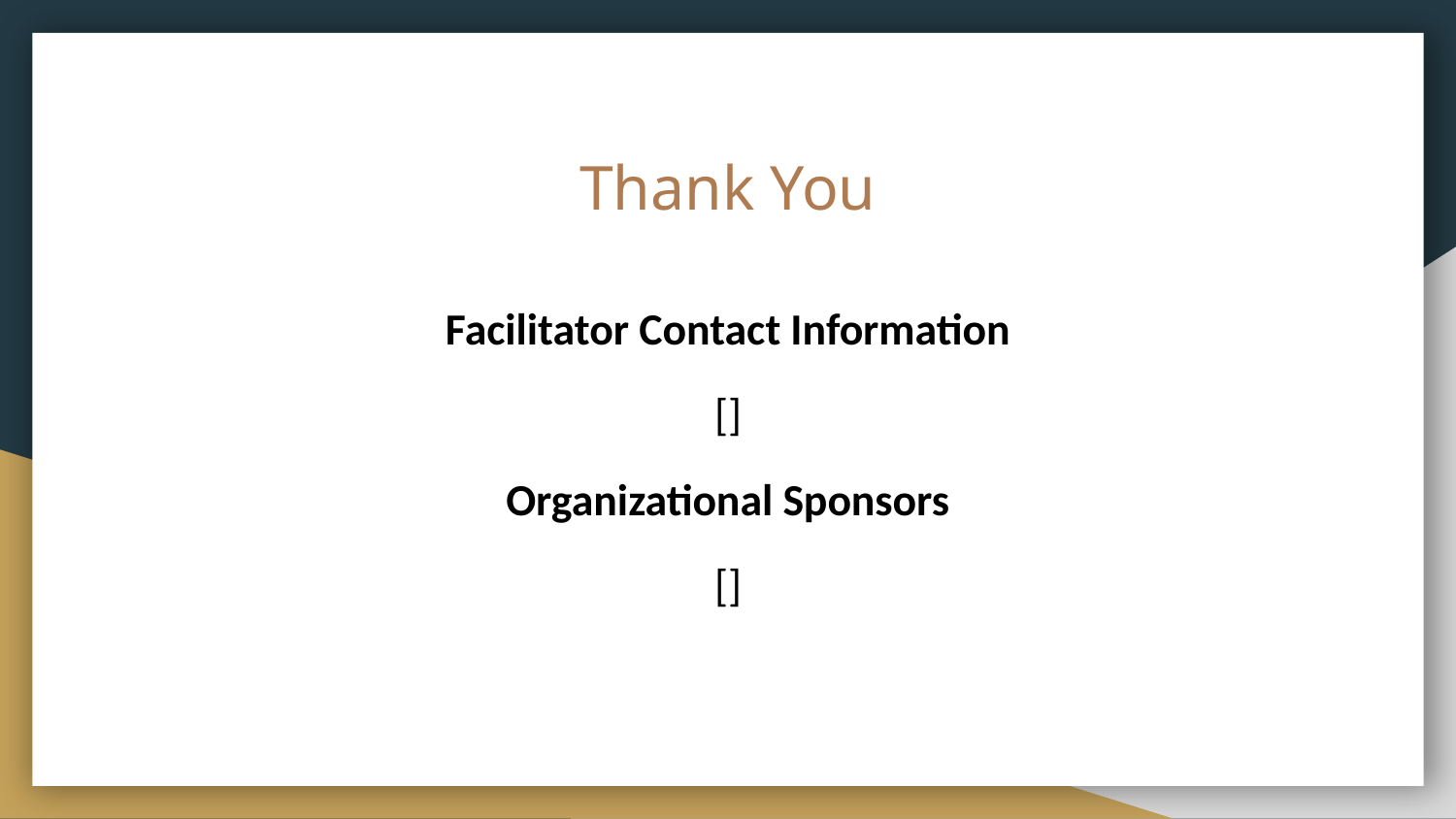

# Thank You
Facilitator Contact Information
[]
Organizational Sponsors
[]
