# Supplementary material for: An Introduction to Traditional Healing in American Indian and Alaska Native Communities
Source: MedEdPORTAL. 2025 Mar 7;21:11506. doi: 10.15766/mep_2374-8265.11506 (PMC11885593; doi:10.15766/mep_2374-8265.11506)
Supplement: Supplementary file 1 — Facilitator Guide.docxInstructional Slides.pptxTrainee Presurvey.docxTrainee Postsurvey.docx [file mep_2374-8265.11506-s001.zip › C. Trainee Presurvey.docx]

Which categories describe you? (choose all that apply)

- American Indian or Alaska Native (e.g. Navajo Nation, Blackfeet Tribe, Mayan, Aztec, Native Village of Barrow, Inupiat Traditional Government, Nome Eskimo Community)
- Asian (e.g. Chinese, Filipino, Asian Indian, Vietnamese, Korean, Japanese)
- Black or African American (e.g. Jamaican, Haitian, Nigerian, Ethiopian, Somalian)
- Hispanic, Latino, or Spanish Origin (e.g. Mexican or Mexican American, Puerto Rican, Cuban, Salvadoran, Dominican, Columbian)
- Middle Eastern or North African (e.g. Lebanese, Iranian, Egyptian, Syrian, Moroccan, Algerian)
- Native Hawaiian or Other Pacific Islander (e.g. Native Hawaiian, Samoan, Chamorro, Tongan, Fijian, Marshallese)
- White (e.g. German, Irish, English, Italian, Polish, French)
- Prefer to self-describe
- Prefer not to say

How do you self-identify? Please check all that apply.

- Gender non-binary, gender non-conforming, genderqueer
- Man
- Woman
- Transgender
- Prefer to self-describe
- Prefer not to say

Select your age range (mark one):

- 24 or below
- 25-29
- 30 - 34
- 35 - 44
- 45 - 54
- 55- 65
- > 65

Select your current professional role (mark one):

- Medical Student
- Medical Resident
- Medical Fellow
- Academic Faculty (MD, DO, NP, PA, PhD, Other)
- Clinician in Non-Academic Practice (MD, DO, NP, PA, Other)
- Other

Are you a first-generation collegiate and/or medical student?

- Yes
- No

How do you identify your sexual orientation? (choose all that apply)

- Asexual
- Bisexual
- Gay
- Lesbian
- Straight/Heterosexual
- Prefer to self-describe
- Prefer not to say

Please rate how much CONFIDENCE do you have in your ability to:

**Learning Objectives**

Describe why traditional healing practices are important in American Indian and Alaska Native communities

- 1, Not confident
- 2,
- 3,
- 4,
- 5, Completely confident

Describe the literature that supports the integration of traditional healing practices with medical services in American Indian and Alaska Native communities

- 1, Not confident
- 2,
- 3,
- 4,
- 5, Completely confident

Identify demonstration projects that assess the impact of traditional healing practices on health outcomes in American Indian and Alaska Native communities

- 1, Not confident
- 2,
- 3,
- 4,
- 5, Completely confident

**Knowledge Questions**

How knowledgeable are you regarding the Indian Health Service and its responsibilities?

- 1, Not at all knowledgeable
- 2
- 3
- 4
- 5, Very knowledgeable

Can Indigenous people receive traditional healing from a(n accredited) healthcare facility?

- Yes
- No
- Unsure

How comfortable do you feel discussing traditional healing practices in coordination with western healthcare with Indigenous patients?

- 1, Not at all comfortable
- 2
- 3
- 4
- 5, Very comfortable

Is there an evidence base to support incorporation of traditional healing into western health care?

- Yes
- No
- Unsure
