# Supplementary material for: An Introduction to Traditional Healing in American Indian and Alaska Native Communities
Source: MedEdPORTAL. 2025 Mar 7;21:11506. doi: 10.15766/mep_2374-8265.11506 (PMC11885593; doi:10.15766/mep_2374-8265.11506)
Supplement: Supplementary file 1 — Facilitator Guide.docxInstructional Slides.pptxTrainee Presurvey.docxTrainee Postsurvey.docx [file mep_2374-8265.11506-s001.zip › D. Trainee Postsurvey.docx]

Please rate how much CONFIDENCE do you have in your ability to:

**Learning Objectives**

Describe why traditional healing practices are important in American Indian and Alaska Native communities

- 1, Not confident
- 2,
- 3,
- 4,
- 5, Completely confident

Describe the literature that supports the integration of traditional healing practices with medical services in American Indian and Alaska Native communities

- 1, Not confident
- 2,
- 3,
- 4,
- 5, Completely confident

Identify demonstration projects that assess the impact of traditional healing practices on health outcomes in American Indian and Alaska Native communities

- 1, Not confident
- 2,
- 3,
- 4,
- 5, Completely confident

**Knowledge Questions**

How knowledgeable are you regarding the Indian Health Service and its responsibilities?

- 1, Not at all knowledgeable
- 2
- 3
- 4
- 5, Very knowledgeable

Can Indigenous people receive traditional healing from a(n accredited) healthcare facility?

- Yes
- No
- Unsure

How comfortable do you feel discussing traditional healing practices in coordination with western healthcare with Indigenous patients?

- 1, Not at all comfortable
- 2
- 3
- 4
- 5, Very comfortable

Is there an evidence base to support incorporation of traditional healing into western health care?

- Yes
- No
- Unsure
